# Cross-neutralization and antigenic characterization of simian and equine group A rotaviruses

**DOI:** 10.1128/jvi.00199-26

**Published:** 2026-03-31

**Authors:** Shalini Soni, Lianne G. Eertink, Ni Shuisong, Alan Loynachan, Samantha M. Barnum, Emma N. Adam, Michael A. Kennedy, Dan Wang, Feng Li

**Affiliations:** 1 Maxwell H. Gluck Equine Research Center, Department of Veterinary Science, University of Kentucky 4530, Lexington, Kentucky, USA; 2 Department of Chemistry and Biochemistry, Miami University 6403, Oxford, Ohio, USA; 3 University of Kentucky Veterinary Diagnostic Laboratory, University of Kentucky 675414, Lexington, Kentucky, USA; 4 Department of Medicine and Epidemiology, School of Veterinary Medicine, University of California 70733, Davis, California, USA; University of Michigan Medical School, Ann Arbor, Michigan, USA

**Keywords:** equine rotavirus A, simian rotavirus SA11, VP8* protein, neutralization, vaccine

## Abstract

Rotaviral diarrhea in foals is caused by two dominant G3P[12] and G14P[12] genotypes of equine rotavirus A (ERVA). Protection of foals against ERVA infection and disease is achievable by passive antibody transfer through colostrum from mares receiving inactivated monovalent G3P[12] vaccine. Simian rotavirus SA11 G3P[2] is also included in ERVA vaccines to protect foals. Here, we characterized cross-neutralization and protection among three distinctive rotaviruses carrying the same or different G (defined by VP7) or P (defined by VP4) genotype by using a rabbit and equine monospecific antisera panel coupled with structural and computational analyses of viral epitopes. Data from these experiments demonstrated that (i) rSA11 G3P[2] and equine G3P[12] neutralized well with each other in rabbit antisera but with less cross-neutralization capacity against equine G14P[12]; (ii) interestingly, rabbit antisera to equine G14P[12] provided more robust cross-protection against rSA11 G3P[2] than the reverse; (iii) despite only a few amino acid differences in VP8* between two equine viruses, rabbit antisera to VP8* of equine G3P[12] moderately cross-neutralized G14P[12] but with potent neutralizing activity against rSA11 G3P[2]; (iv) surprisingly, the breadth and magnitude of cross-neutralization among three viruses in equine antisera were broader and stronger than those observed in rabbit antisera; and (v) multiple highly solvent-exposed amino acid residues conserved in VP7 and VP8* among three viruses may serve as novel B cell epitopes that contribute to the observed cross-neutralization. Taken together, these results highlight that cross-neutralization determinants among different rotaviruses are complex and dependent on specific viral context and animal species.

**IMPORTANCE:**

Our findings support that the G genotype determined by the VP7 protein plays a more important role, within the virus panel examined in this study, in determining broad neutralization specificity than previously thought. Our work has implications for vaccine design, suggesting that the inclusion of diverse G genotypes may be necessary to achieve broader protection. The P genotype, determined by the VP4 protein, exhibited variable levels of cross-neutralization, indicating that the P genotype alone may not be sufficient to induce strong cross-neutralization among different rotaviral strains. G genotype- or VP7-driven cross-protection may be conferred by novel epitopes identified structurally in this study. Finally, our study revealed that horse sera were more capable of cross-neutralizing different equine and simian rotaviruses than rabbit sera. This finding has implications for vaccine development, underscoring the need to evaluate candidate vaccines across multiple species or in species-relevant models to ensure broad and effective vaccine-mediated protection against rotavirus infection.

## INTRODUCTION

Rotaviruses (RVs) of animals and poultry have been classified into multiple groups based on the antigenic differences of the middle-layer VP6 protein. Among them, group A RV (RVA) is more prevalent and infects a wide variety of hosts, including humans, equines, pigs, bovines, and simians. Equine RVA (ERVA) is the leading cause of diarrhea in foals under 6 months of age (1). Foals born to unvaccinated mares can show signs of infection as early as 12 h of age (2). However, foals from vaccinated mares are most vulnerable between 3 and 4 months of age when maternal antibodies wane to low levels (3). Since the discovery of ERVA in the United Kingdom in 1975 (4), many cases of diarrhea associated with ERVA infection have been reported worldwide (5–8). ERVA is highly contagious and spreads quickly among foals via the fecal-oral route and can lead to severe and potentially fatal diarrhea if not immediately and properly treated (5, 9). RVA infections are also a major concern in humans, as children are at risk of infection. Due to the virus’s segmented nature in the genome that allows for reassortment, a newly formed reassortant has the potential to transmit across various species, which can pose a health risk to mammals, including humans who do not have preexisting immunity (5). Between 2013 and 2019, reassortment of RVA led to outbreaks of severe gastroenteritis and hospitalizations of children. This was due to a novel reassortment event involving equines, humans, and potentially other animals, leading to the generation of a novel equine-like G3 RVA (10, 11).

RV particles are triple-layered, non-enveloped, and contain a segmented genome of 11 double-stranded RNA segments, which encode six structural proteins (VP1–4, VP6, and VP7) and six non-structural proteins (NSP1–NSP6) (5, 12, 13). Antigenic and sequence differences in the middle layer capsid protein, VP6, form the basis for classifying RVs into 11 groups (Groups A–D and F–L). Furthermore, RVAs can be further differentiated into G and P genotypes with a dual nomenclature system based on the sequence and antigenic variations of the two outer capsid proteins VP7 and VP4 (14–16). There are 36 G and 51 P RVA genotypes reported to date, and different combinations of strains are found in humans and animals due to frequent reassortment events (14). Since VP4 and VP7 are located at the surface of the viral particles, they are primary targets of host immune responses, and antibodies to both VP4 (VP8* and VP5*) and VP7 can neutralize RVs (5, 17). Until now, in horses, six G-types (G3, G14, G5, G8, G10, and G13) and six P-types (P[1], P[3], P[7], P[11], P[12], and P[18]) have been reported (5, 18–26) with several combinations, such as G3P[3], G3P[12], G14P[12], G5P[7], G8P[1], G10P[11], and G13P[18]. Among these, G3P[12] and G14P[12] genotypes are widespread and epizootic in horse populations worldwide (8, 27–29).

Protection of foals against RV infection and disease has a high dependence on passive antibody transfer through colostrum from the vaccinated mares (30–32). However, the concentration of these colostrum-derived antibodies decreases immediately after birth, and antibody levels reach their lowest effective threshold at approximately 3–4 months of age, thereby making these foals prone to RVA infection (3). Currently, there are three main types of inactivated equine RV vaccines developed for use in different regions of the world, primarily based on the G3P[12] strain. In the USA, an inactivated cell-culture-adapted H2 strain (G3P[12]) is used as a monovalent vaccine, which is also used in several other countries, including New Zealand, Australia, and Europe. Since 1996, Argentina has used a trivalent vaccine formulation that contains simian RVA SA11 (G3P[2]), bovine RVA NCDV-Lincoln (G6P[1]), and equine RVA H2 (G3P[12]). This vaccine has considerably reduced RVA-associated diarrhea in foals (5, 22, 30, 31). Additionally, in Japan, an inactivated vaccine based on the prevalent equine RVA HO-5 strain (G3BP[12]) has been used, leading to improved protection of foals against infection (33). Because most vaccines target the G3P[12] strain specifically for stimulating protective antibody responses, infection with the G14P[12] strain has slightly increased in frequency over time and shows a cyclic pattern from year to year in foals (34, 35). In central Kentucky, RVA outbreaks in foals born to vaccinated mares remain a concern for the equine industry (29). Although the use of a monovalent vaccine has reduced disease severity, breakthrough variants of the homologous G3P[12] genotype and heterologous G14P[12] strains are observed on some occasions (30, 31, 33). In addition, clinical studies suggest partial cross-protection by G3P[12] antibodies against G14P[12], but virus neutralization titers against the heterologous strains are moderately lower than those for the homologous strains (3, 36).

To understand the importance of including other RVA strains, such as the simian SA11 strain, alongside equine strains to enhance cross-protection against diverse RVA strains in equines, including G14P[12], which continues to present challenges for vaccine-mediated control, we investigated rSA11 strain-mediated antibody responses against both the G3 and G14 equine genotypes. rSA11 is known for its high growth capacity in cell culture, robust immunogenicity, and ability to elicit heterotypic antibody responses (37, 38). It has been extensively used in experimental vaccines and immunogenicity studies as a model strain because of its well-characterized genetics, structure, and receptor interactions. Both VP4 and VP7 contain multiple discrete B cell epitopes, which are major targets of neutralizing antibodies (39–41). Several studies have found that a single amino acid substitution at position 150 in VP8* within an epitope site can alter loop conformation and antibody recognition (42, 43).

Including additional RVA strains could elicit broader neutralizing antibody (NAb) responses, making them useful vaccine components. While both equine G3P[12] and rSA11 G3P[2] share the same G3 genotype (VP7), they differ in VP4 specificity. VP4 is the determinant of the P genotype and a key target of neutralizing antibodies. In contrast, equine G14P[12] and G3P[12] strains share the same P genotype (VP4), with the minimal sequence variation in VP8* and VP5* epitope sites but differ in G genotype (VP7) (8). Despite the antigenic similarities in VP4, cross-protection between G3P[12] and G14P[12] is often variable in the field (3). Based on previous immunological studies, which are largely based on mice, the VP7 protein is more immunogenic than the VP4 protein, and such antigenic distinction has led to the recognition of VP7 as the major antigen and VP4 as the minor antigen, despite both being targets of virus-neutralizing antibody responses (44). It remains unclear whether there are some notable discrepancies among different host species in terms of the breadth and magnitude of VP7- or VP4-specific antibodies in neutralizing different rotaviruses.

In this study, we investigated the virus-neutralizing antibody responses elicited by rabbits and horses immunized with rSA11 G3P[2], Eq-ref-G3P[12], and rabbits immunized with Eq-ref-G14P[12] RVA strains, as well as by rabbits immunized with the recombinant VP8* protein of Eq-ref-G3P[12]. Furthermore, we immunized foals born to mares that were not vaccinated during their gestation periods to evaluate the antibody response to the G3P[12] monovalent vaccine (H2 strain) and the cross-protection against heterologous Eq-ref-G14P[12]s and rSA11 G3P[2] strains. Our cross-neutralization experiments were designed to explore how antibodies induced by different RVA strains or subunit antigens recognize and neutralize homologous and heterologous strains. The other goal of this work was to better understand the importance of a few substitutions, particularly within epitope sites, in potentially altering the cross-genotype neutralization response. We also investigated whether the specificity and the magnitude of anti-RVA-neutralizing antibody responses varied among different host species. The results of our experiments provide novel insights into the cross-neutralization mechanism among different RVA genotypes, which should inform strategies for designing vaccines that achieve broader protection against circulating ERVA strains in horses and agricultural animals.

## RESULTS

### Rabbit simian rSA11 G3P[2] serum is more effective in neutralizing equine G3P[12] than equine G14P[12]

**Fig 1:**
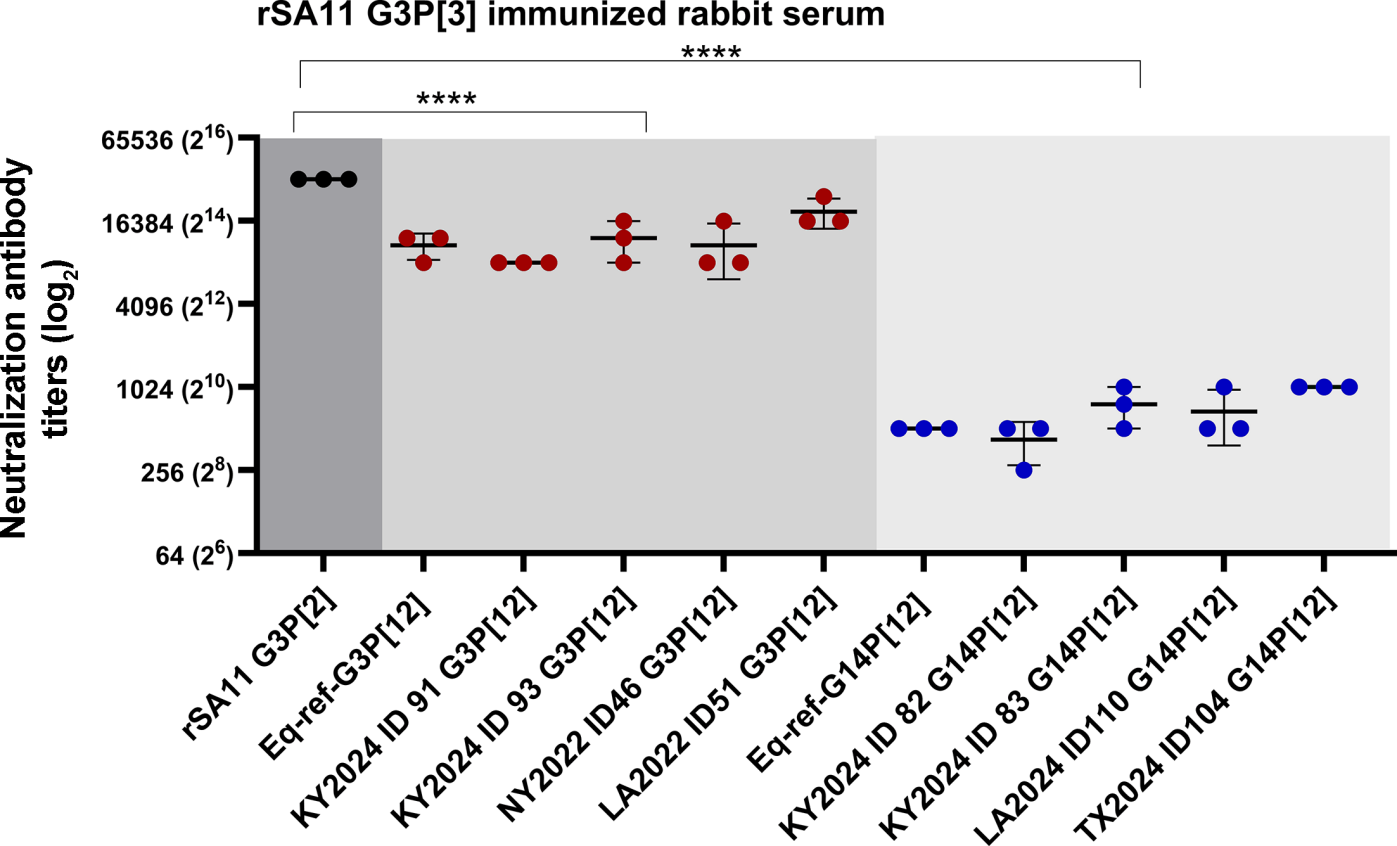
Neutralization antibody response of rSA11 immunized rabbit serum. The serum from rabbits immunized with rSA11 G3P[2] strain was tested for its ability to neutralize rotavirus strains: the homologous simian SA11 (rSA11), a reference equine Eq-ref-G3P[12], a reference equine Eq-ref-G14P[12], and eight field strains (ID 46, 51, 91, 93, 82, 83, 101, and 104). Neutralization antibody titers are plotted on a logarithmic (log_2_) scale. Each point represents the mean of duplicate measurements from three independent experiments; horizontal bars indicate the mean ± standard deviation. Significant difference was observed between rSa11 and G3 Ref, and between rSA11 and G14 Ref (****, *P* < 0.0001; two-way ANOVA, Dunnett’s multiple comparisons test).

To understand the potential impact of incorporating the rSA11 G3P[2] strain into the equine RV vaccination regime to protect foals from RV infection, we generated rabbit serum against the simian rSA11 G3P[2] strain (rescued by SA11 reverse genetics system) and determined its cross-neutralization against two equine genotypes sharing the same P genotype but with different G genotypes, G3P[12] and G14P[12], which predominantly circulate in equines. The neutralization experiments were conducted using MA-104 (Rhesus monkey kidney epithelial cells). The rSA11 strain used in this study belongs to the G3P[2] genotype. Two equine strains (G3P[12] and G14P[12]) are well characterized and have been previously published as reference strains in our laboratory (8). As anticipated, the rSA11 G3P[2] rabbit serum showed strong neutralization titers against the homologous RV strain, rSA11, with a mean NAb titer of 32,778, indicating a robust strain-specific neutralizing antibody response (Fig. 1). We also observed that rSA11 G3P[2] rabbit serum effectively cross-neutralized equine G3P[12], with a mean NAb titer of 10,925. A potent cross-neutralization between rSA11 G3P[2] and equine G3P[12] likely can be attributed to the overlap of antigenic epitope sequences residing in the VP7 protein, consistent with the shared G3 genotype in the rSA11 G3P[2] and equine G3P[12] strains. In contrast, rSA11 G3P[2] rabbit serum showed decreased neutralizing antibody titers against equine G14P[12], with an approximate 21-fold reduction in NAb titers compared to the homologous strain titers, with a mean NAb titer of 520. This result indicated a lower similarity of antigenic sites in the VP7 protein between rSA11 G3P[2] and equine G14P[12] (Fig. 1). The observed differential cross-neutralization activity between G3P[12] and G14P[12] with rabbit rSA11 G3P[2] serum appears to support a theme that RVs with the same G genotype type, defined by the VP7 protein (the major antigen), can more effectively cross-neutralize each other than RVs with different P genotypes defined by the VP4 protein (the minor antigen).

To further determine whether rSA11 G3P[2] rabbit serum can cross-neutralize ERVA contemporary field strains in a fashion similar to those observed with the reference strains, we included a panel of eight ERVA field isolates in our experiment. These strains were isolated from diseased foals with clinical diarrhea in Kentucky, New York, Texas, and Louisiana in 2024 and 2022: four G3P[12] strains (strain IDs: KY2024 ID91, KY2024 ID93, NY2022 ID46, and LA2022 ID51) and four G14P[12] strains (strain IDs: KY2024 ID 82, KY2024 ID 83, LA2024 ID101, and TX2024 ID104). Neutralization titers against these contemporary strains showed comparable results and trends to the corresponding reference strains (Fig. 1). Taken together, using this panel of viruses, including both reference strains and contemporary field strains, we showed the cross-neutralizing activities conferred by rSA11-induced antibodies against ERVA strains of epidemiological relevance. Our data further demonstrated that the more potent cross-neutralization occurs between rSA11 and equine RVs bearing the same G genotype as rSA11 (i.e., G3).

### Rabbit equine G3P[12] serum potently neutralizes simian rSA11 G3P[2] but has a low neutralization activity against equine G14P[12]

**Fig 2:**
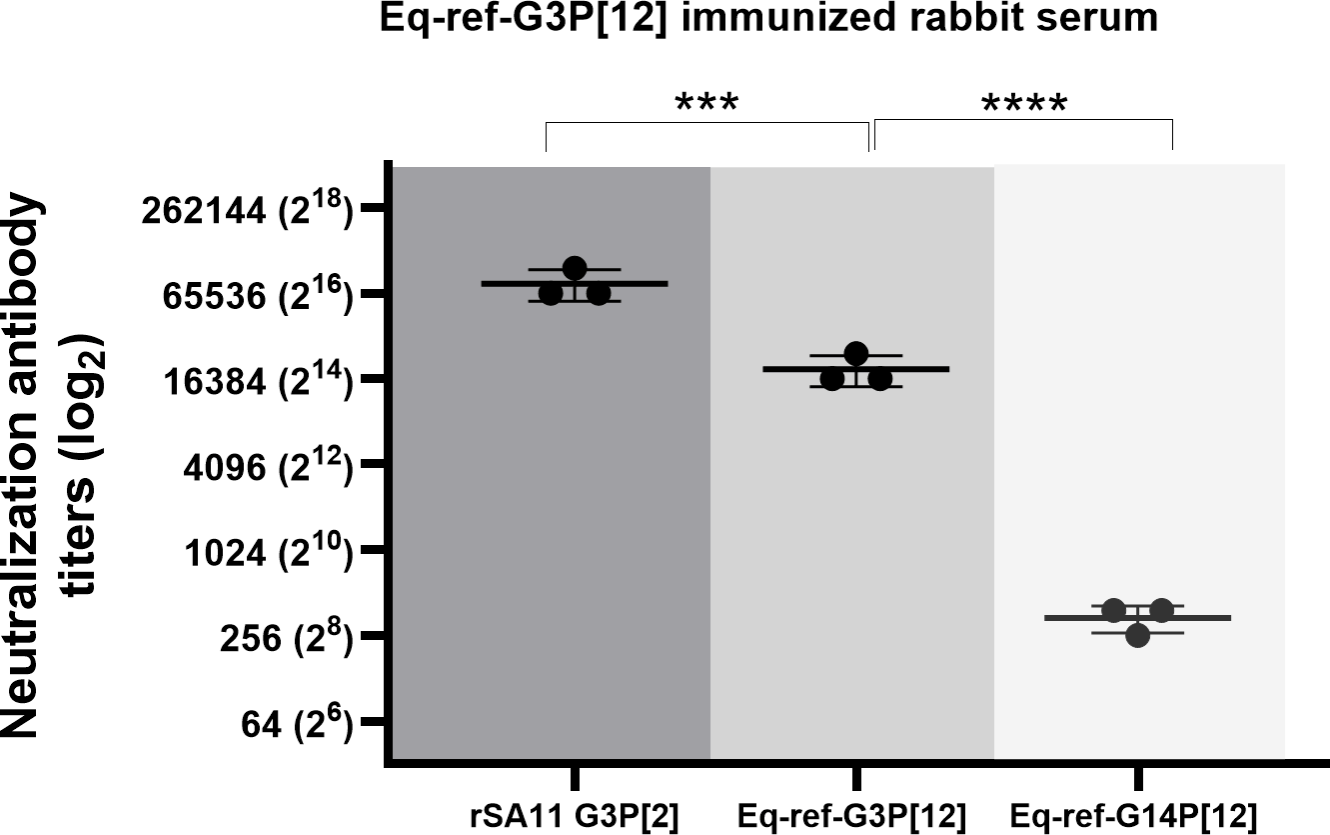
Neutralization antibody response of Eq-ref-G3P[12] immunized rabbit serum. The serum from the rabbit immunized with Eq-ref-G3P[12] strain was tested for its ability to neutralize RV strains: the homologous simian rSA11, a reference equine Eq-ref-G3P[12], and reference equine Eq-ref-G14P[12]. Neutralization antibody titers are plotted on a logarithmic (log_2_) scale, while each point represents the mean of duplicate measurements from three independent experiments; horizontal bars indicate the mean ± standard deviation. Significant differences were observed between rSA11 and G3 Ref (***, *P* < 0.0004) and between G3 Ref and G14 Ref (****, *P* < 0.0001; two-way ANOVA Dunnett’s multiple comparisons test).

To determine the potential two-way cross-neutralization between equine G3P[12] and simian rSA11 G3P[2] and further define whether RVs with the same P genotype (defined by the VP4 protein) can undergo cross-neutralization, we generated rabbit serum against the equine G3P[12] reference strain and characterized its ability to cross-neutralize both homologous and heterologous strains. In this experiment, we included three RV strains: rSA11 G3P[2], Eq-ref-G3P[12], and Eq-ref-G14P[12] (Fig. 2). As expected, we found high NAb titers against the homologous equine strain with a mean NAb titer of 19,115. Interestingly, the mean NAb titer against the rSA11 G3P[2] strain was 76,459, which was approximately fourfold higher than that of the homologous Eq-ref-G3P[12] strain. The higher NAb titer than that observed against the homologous strain may depend on several factors, such as the sensitivity of a strain to neutralization, VP7 trimer structural stability, and subtle differences in VP4 interactions with other structural proteins, such as VP4 and VP6 that may change virion dynamics and its response to NAb epitope accessibility and strain-specific replication efficiency (45–47). Additionally, SA11 is a highly cell culture-adapted strain. These factors may influence the NAb titers that may give rise to the data in Fig. 2. In contrast, the NAb response against the Eq-ref-G14P[12] strain was significantly lower with a mean NAb titer of 341 (Fig. 2). Overall, these findings highlight that the antibody response against the same G genotype exhibits high two-way cross-reactivity between rSA11 G3P[2] and Eq-ref-G3P[12] probably due to shared VP7 (G3)-specific epitopes. However, the cross-reactivity between two ERVAs with the same P genotype (i.e., VP4 protein) was much lower, further elucidating the role of VP7 protein (G type) in cross-neutralizing a heterologous virus with the same P genotype.

### Rabbit Eq-ref-G14P[12] serum effectively neutralizes simian rSA11 G3P[2] with a moderate ability to neutralize Eq-ref-G3P[12] sharing the same P genotype

**Fig 3:**
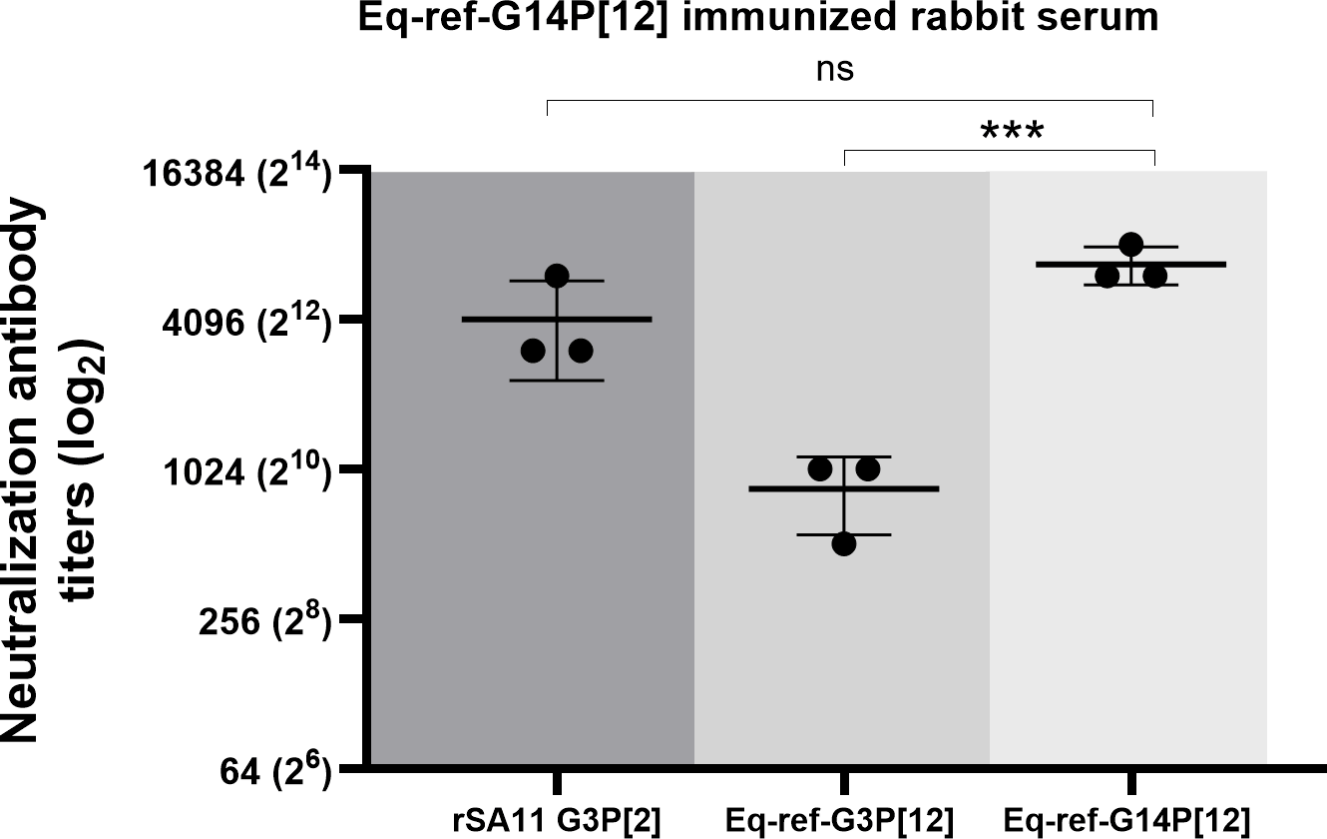
Neutralization antibody response of Eq-ref-G14P[12] immunized rabbit serum. The serum from the rabbit immunized with Eq-ref-G14P[12] strain was tested for its ability to neutralize RV strains: the homologous simian rSA11, a reference equine Eq-ref-G3P[12], and reference equine Eq-ref-G14P[12]. Neutralization titers are plotted on a logarithmic (log_2_) scale, while each point represents the mean of duplicate measurements from three independent experiments; horizontal bars indicate the mean ± standard deviation. No significant difference (ns) was observed between G14 Ref and rSA11, whereas titers against G3 Ref were significant (***, *P* < 0.0007; two-way ANOVA).

Next, we were interested in investigating the neutralizing antibody response generated in rabbits against the Eq-ref-G14P[12] strain and determining whether it would possess measurable neutralizing activities against the heterologous equine Eq-ref-G3P[12] and rSA11 G3P[2] strains. For this, the same panel of viruses was used (Fig. 3). The serum NAb titer against the immunized homologous equine Eq-ref-G14P[12] virus was the highest, with a mean NAb titer of 6,829. Interestingly, the NAb titer observed against rSA11 G3P[2] was comparable to Eq-ref-G14P[12], with the mean titer of 4,097, which indicated possible antigenic similarities despite both viruses carrying different G and P genotypes. Notably, the rabbit rSA11 G3P[2] serum exhibited only a weak neutralization against Eq-ref-G14P[12] (Fig. 2). The combination of these data indicates a one-way cross-protection in that Eq-ref-G14P[12] serum provides robust cross-protection against the rSA11 G3P[2], whereas rSA11 G3P[2] serum confers limited protection against the Eq-ref-G14P[12]. Furthermore, as shown in Fig. 3, Eq-ref-G14P[12] serum poorly neutralized the heterologous strain Eq-ref-G3P[12] with a mean NAb titer of 853, an eightfold reduction relative to the homologous Eq-ref-G14P[12] strain. Mutually poor cross-neutralization between Eq-ref-G3P[12] and Eq-ref-G14P[12] with the rabbit sera indicates that these two viruses are antigenically distinct, despite sharing the same P genotype. These findings further implicate that the observed antigenic difference is largely driven by the VP7 protein (i.e., G genotype).

It is intriguing to note that, despite the differences between G and P genotypes, Eq-ref-G14P[12] serum potently neutralized rSA11 G3P[2] virus, despite this cross-neutralization not being observed in the opposite direction. This one-way cross-neutralization supports the notion that Eq-ref-G14P[12] may exhibit relatively broad antigenicity related to rSA11 virus, which allows the virus to elicit antibodies that can neutralize rSA11 G3P[2] carrying different G and P genotypes. Despite this profound cross-neutralization between Eq-ref-G14P[12] and rSA11 G3P[2], rabbit Eq-ref-G14P[12] serum remains less effective in neutralizing Eq-ref-G3P[12] sharing the same P genotype, which is consistent with our previously published data (8). The lack of substantial rabbit antibody-mediated cross-protection between these two equine genotypes in both directions further highlights that the amino acid differences in the VP7 protein (defining the G genotype) are responsible for the antigenic distance observed in the context of rabbit immunity.

### Rabbit P[12]-VP8* serum shows a potent neutralizing activity against Eq-ref-G3P[12] and rSA11 G3P[2] with a reduced neutralization against Eq-ref-G14P[12]

**Fig 4:**
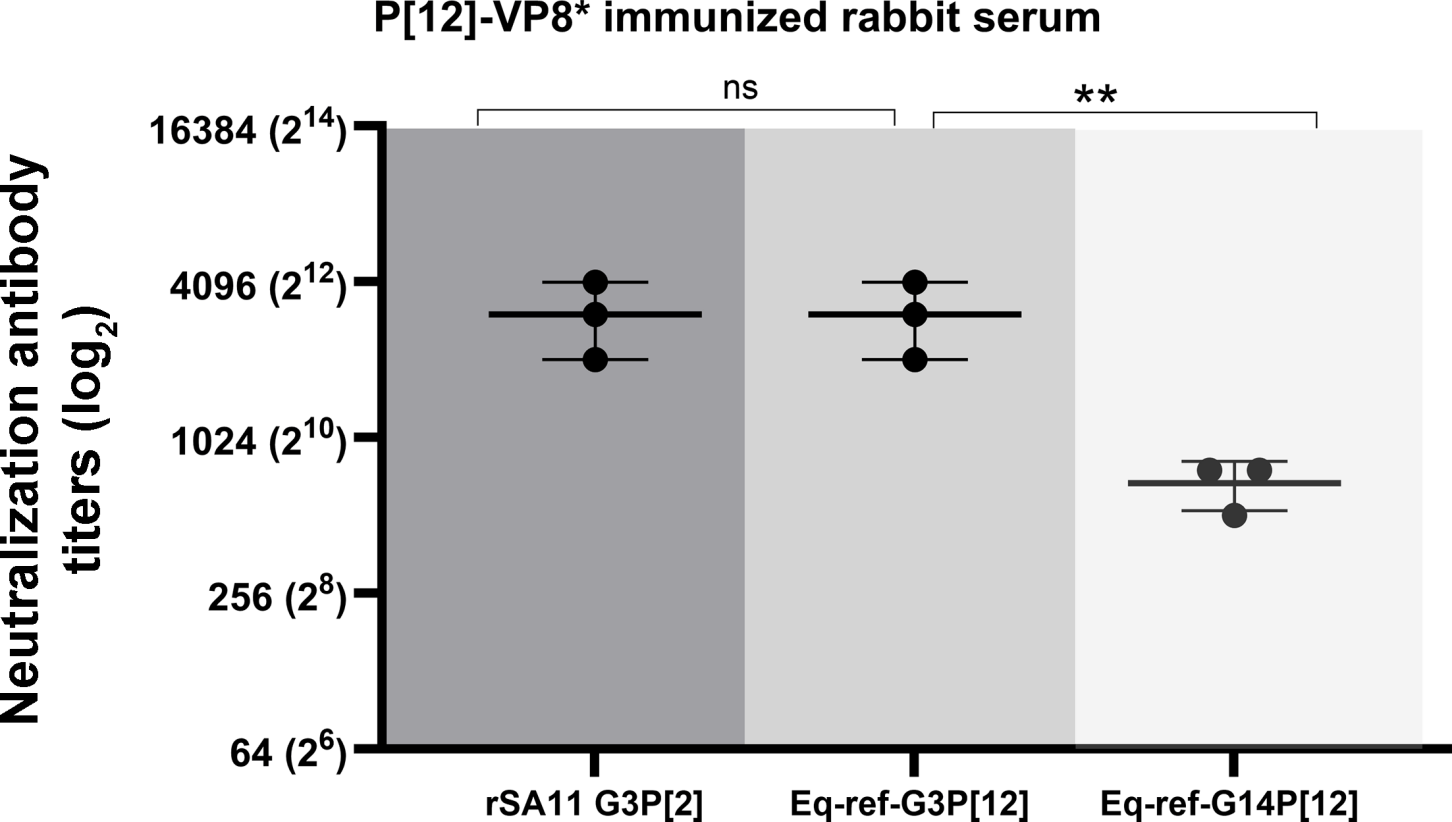
Neutralization antibody response of P[12]-VP8* immunized rabbit serum. The serum from the rabbit immunized with P[12]-VP8* was tested for its ability to neutralize three RV strains: the simian rSA11, a reference equine G3P[12], and reference equine G14P[12]. Neutralization titers are plotted on a logarithmic (log_2_) scale, while each point represents the mean of duplicate measurements from three independent experiments; horizontal bars indicate the mean ± standard deviation. No significant difference (ns) was observed between rSA11 and G3 Ref, whereas titers against G14 Ref were significant (**, *P* < 0.0075; two-way ANOVA).

Our overall results generated so far suggest that the higher neutralization responses observed may be attributed to the shared G genotype, which is determined by the VP7 protein, whereas VP4 (VP8* and VP5*) appears to play a minor role in conferring the cross-neutralization. To further investigate this phenomenon, we immunized rabbits with the purified P[12] -VP8* protein, and the resulting sera were used to evaluate the neutralization response (Fig. 4). As expected, the P[12]-VP8*-immunized rabbit serum showed a high NAb titer against the homologous Eq-ref-G3P[12] strain, with a mean NAb titer of 3,072 (Fig. 4). In contrast, the NAb titer against the heterologous Eq-ref-G14P[12] strain was 4.4-fold lower, with a mean NAb titer of 683. The VP8* epitope sequences between these two equine strains differ only by two amino acids, and these data suggest that even a few changes in the epitope can affect the antibody response. Surprisingly, the P[12]-VP8* rabbit serum neutralized the rSA11 G3P[2] strain at levels comparable to those against the homologous Eq-ref-G3P[12], despite the substantial divergence in VP8* sequence between rSA11 G3P[2] and the equine strain. This result further demonstrates that there may be additional, or yet unidentified, amino acid residues in VP8* of equine strains that function as cross-reactive neutralizing epitopes, which possibly enable recognition of antigenically distinct strains such as rSA11 G3P[2], which warrants future investigation.

### Breadth and magnitude of the observed cross-neutralization of rSA11 G3P[2], Eq-ref-G14P[12], and Eq-ref-G14P[12] differ between horses and rabbits

**Fig 5:**
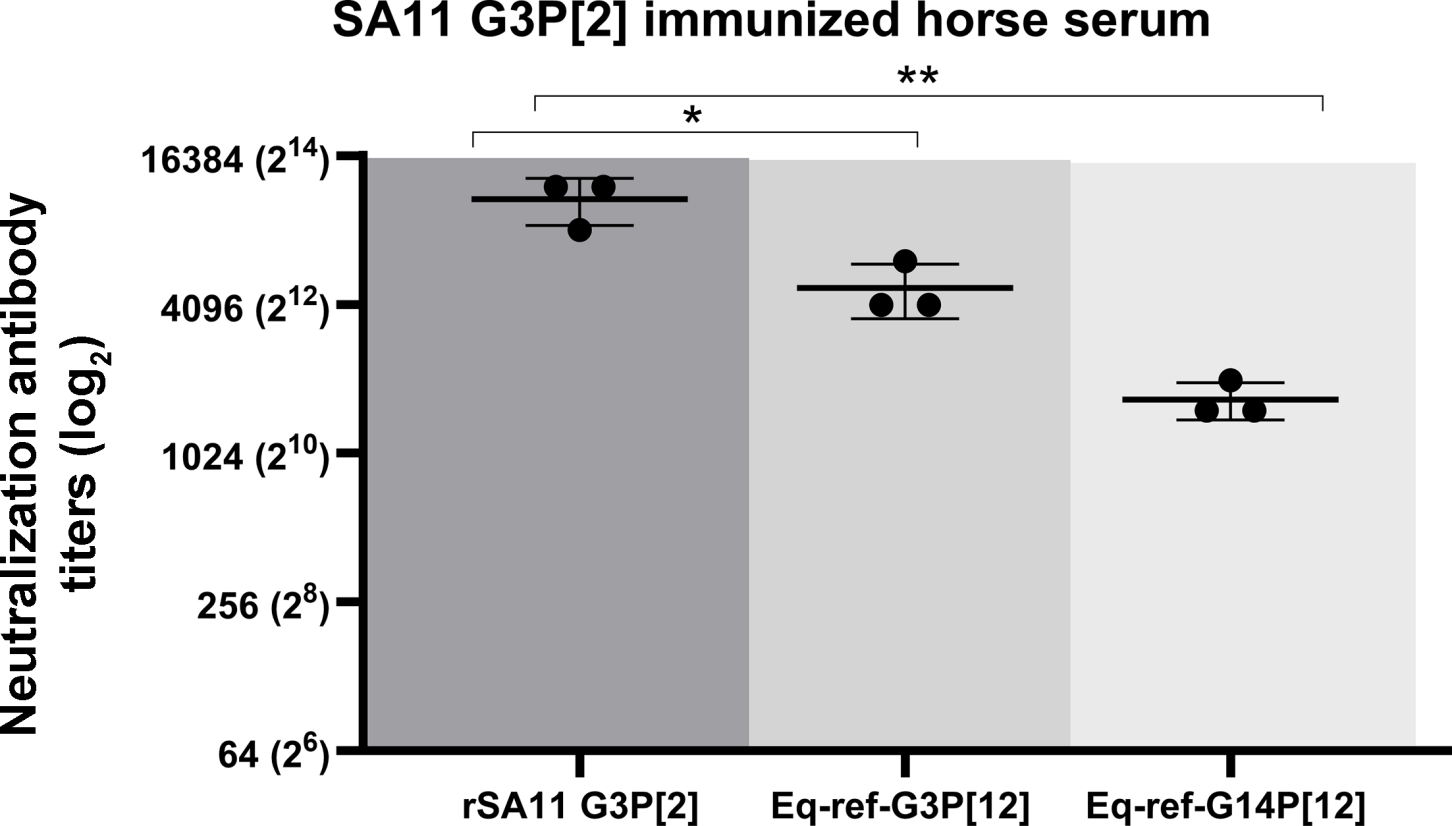
Neutralization antibody response with horse hyperimmune serum raised against SA11 G3P[2]**.** The serum from the horse immunized with the SA11 G3P[2] strain was tested for its ability to neutralize RV strains: the homologous simian rSA11, a reference equine Eq-ref-G3P[12], and reference equine Eq-ref-G14P[12]. Neutralization titers are plotted on a logarithmic (log_2_) scale, while each point represents the mean of duplicate measurements from three independent experiments; horizontal bars indicate the mean ± standard deviation. A significant difference was observed between rSA11 and G3 Ref (*, *P* < 0.215), whereas titers against G14 Ref were also significant (**, *P* < 0.0011; two-way ANOVA).

So far, rabbit rSA11 serum consistently showed high NAb titers, particularly against equine G3P[12] viruses but also demonstrated limited cross-protective response against equine G14P[12] viruses. We next aimed to investigate whether there is a host species-dependent effect on the cross-neutralization of these divergent rotaviruses with the same G or same P genotype. In this context, we leveraged an equine SA11 G3P[2] reference serum and characterized its ability to neutralize the same panel of RVs that was analyzed with rabbit sera (Fig. 1 to 3). As shown in Fig. 5, equine SA11 G3P[2] serum showed the highest NAb titer against the homologous rSA11 G3P[2] strain, with a mean titer of 10,925, which confirmed the strong strain-specific immune response observed in rabbits (Fig. 1). Furthermore, equine SA11 G3P[2] serum showed only a twofold reduced NAb titer against the heterologous Eq-ref-G3P[12] strain, with a mean of 4,779. This twofold reduction in NAb titers between rSA11 G3P[2] and Eq-ref-G3P[12] viruses measured with the horse serum is similar to the response we observed with rabbit rSA11 G3P[2] serum, in that an approximately threefold reduction in NAb titers was observed between these two viruses. This high and species-independent cross-reactivity is likely due to the shared G3 genotype between these two strains, suggesting that the similar VP7 protein provides better cross-neutralizing antibody responses. Furthermore, in contrast to a significant reduction (i.e., 21-fold) in NAb titers against equine G14P[12] when rabbit rSA11 G3P[2] serum was used in the neutralization assay (Fig. 1), equine SA11 G3P[2] serum only showed a moderate decrease in cross-neutralization against Eq-ref-G14P[12], which was about a sixfold reduction in NAb titers with a mean of 1,691 (Fig. 5). The stronger neutralization of Eq-ref-G14P[12] by equine antibodies against rSA11 G3P[2] when compared to the rabbit antibodies indicates that the breadth and magnitude of the NAb response may vary between different host species due to the difference in host-specific immune responses.

Our results using SA11 G3P[2]-immunized equine serum showed higher cross-neutralization breadth against the heterologous G14P[12] strain, whereas rabbit serum showed higher neutralization magnitude against the G3P[12] strain. These data appear to emphasize that host species-specific immunity plays a role in the cross-neutralizing activity among rotaviruses. To further investigate and validate this observation, we took advantage of foal sera generated by the current vaccine strain used in Central Kentucky equine industry, which is based on the monovalent G3P[12] H2 strain, and analyzed their ability to neutralize the same panel of rotaviruses, including Eq-ref-G3P[12], Eq-ref-G14P[12], and rSA11 G3P[2]. For this experiment, we selected three foals for immunization, which was based on the lowest pre-vaccination maternal NAb titers in these animals. All foals had NAb titers below 32 against Eq-ref-G3P[12] and Eq-ref-G14P[12] before vaccination (Fig. S1). Therefore, these foals were most likely to reveal a vaccine-induced antibody response. ERVA maternal neutralizing antibodies start declining immediately after birth, and maternal antibodies are usually at their lowest in foals at the age of 90–120 days (3), creating a window of opportunity for active immunization.

**Fig 6:**
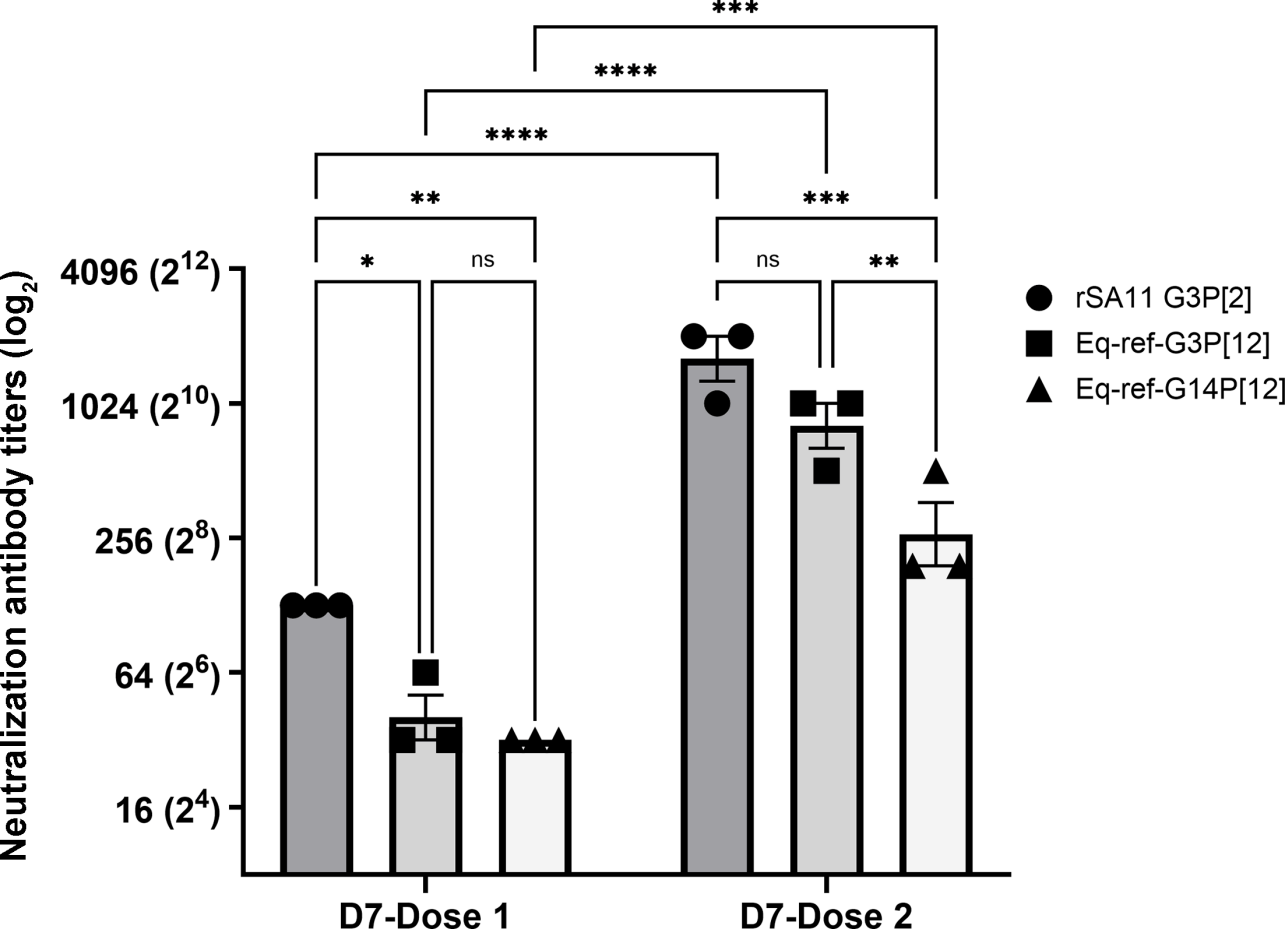
Neutralization antibody response in the serum panel derived from vaccinated foals. The serum samples from vaccinated foals, collected 7 days after the first and second vaccinations, were tested for their ability to neutralize RV strains: the homologous simian rSA11, a reference equine Eq-ref-G3P[12], and reference equine Eq-ref-G14P[12]. Neutralization titers are plotted on a logarithmic (log_2_) scale, while each point represents the mean of duplicate measurements from three independent experiments; horizontal bars indicate the mean ± standard deviation. Statistical analysis was performed using a two-way ANOVA. ns (*P* ≥ 0.05), *P < 0.5, **P < 0.01, ***P < 0.001, ****P < 0.0001.

We evaluated the NAb titers with sera collected from vaccinated foals against Eq-ref-G3P[12], Eq-ref-G14P[12], and rSA11 G3P[2] (Fig. 6). Following the first vaccination, moderate levels of NAb titers were observed against the homologous Eq-ref-G3P[12] strain, with a mean NAb titer of 32 for foals ID04 and ID05, and 64 for foal ID08 (Fig. 6). Furthermore, all three foals showed a mean NAb titer of 32 against the heterologous Eq-ref-G14P[12] strain. NAb titers increased substantially following the second vaccination. Specifically, NAb titers against Eq-ref-G3P[12] rose to 512 in foal ID04 and 1,024 in both foals ID05 and ID08. For Eq-ref-G14P[12], NAb titers increased to 128 in foals ID04 and ID08, and 512 in foal ID05. These increased NAb titers after the second vaccination indicated the active seroconversion following immunization. Similar to the reference strains, comparable NAb titers were observed against diverse field isolates (Fig. S2). Finally, when these sera were analyzed for their ability to neutralize the rSA11 G3P[2] strain, they showed one- to twofold higher neutralization titers against the rSA11 G3P[2] strain using serum collected at 7 days post-first and 7 days post-second vaccination compared to equine strains, with titers increasing over time. Remarkably, this pattern mirrored the response observed with rabbit serum immunized against Eq-ref-G3P[12], as in both species, Eq-ref-G3P[12] serum showed slightly higher NAb titers against rSA11 G3P[2] than against itself. Interestingly, compared to rabbit serum generated with Eq-ref-G3P[12], where a limited cross-neutralization was observed between Eq-ref-G3P[12] and Eq-ref-G14P[12], equine serum derived from foals receiving the G3P[12] H2 immunization only demonstrated an approximately two- to fourfold reduction in NAb titers against Eq-ref-G14P[12] when compared with Eq-ref-G3P[12]. These data suggest that NAb responses are likely species dependent, which may influence different levels of cross-reactivity among different RVs measured when sera from different species are used in the assay.

### Sequence and epitope comparison of VP7 among rSA11 G3P[2], equine G3P[12], and G14P[12] genotypes

**Fig 7:**
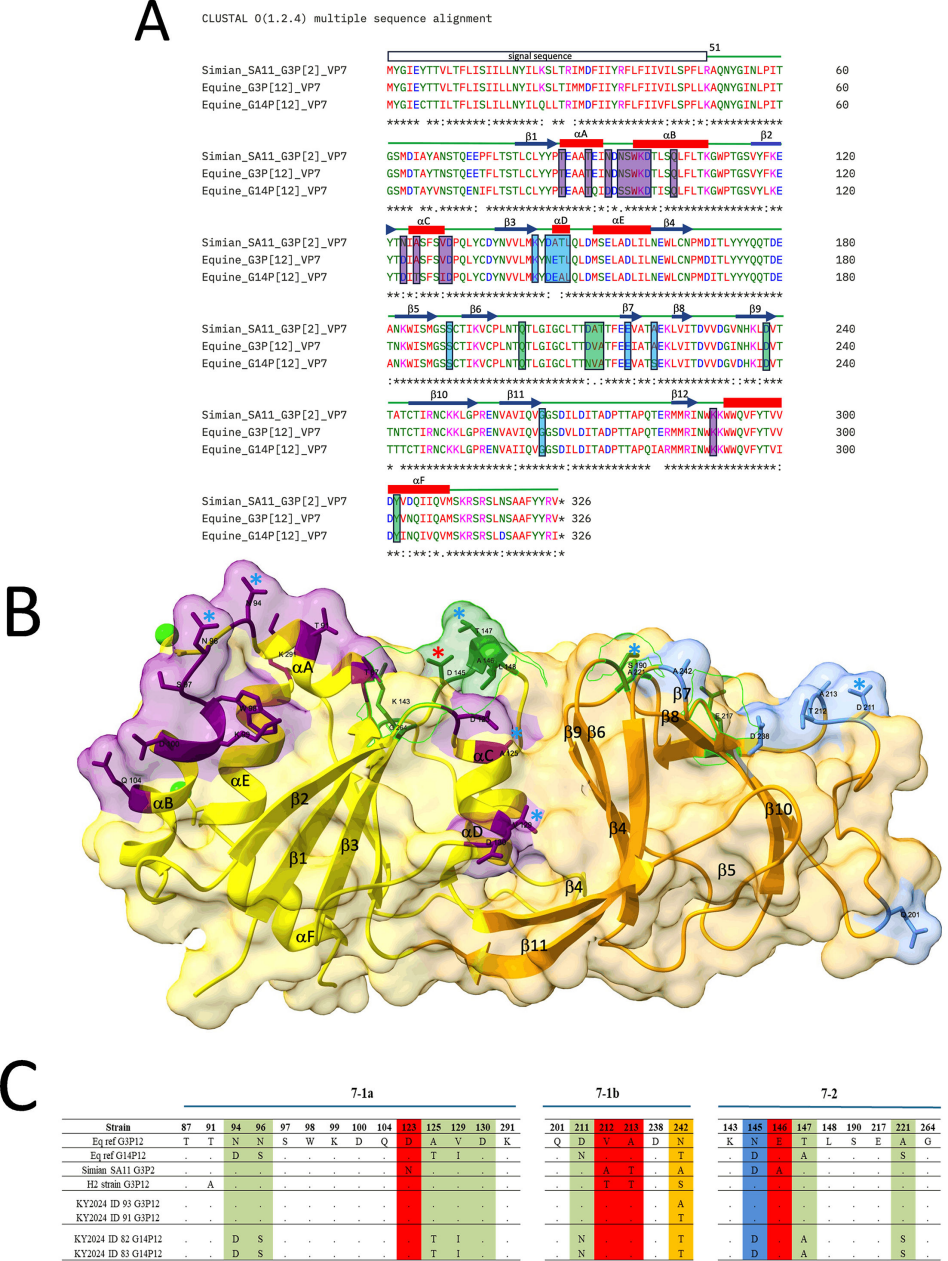
Analysis of amino acid differences in the epitope regions of the simian, equine G3, and equine G14 VP7 sequences. (**A**) Clustal (Omega) multiple sequence alignment using the simian SA11 VP7, equine G3P VP7, and equine G14 VP7 sequences. The secondary structural elements are labeled above the simian sequence. The 7-1a, 7-1b, and 7-2 epitope residues are highlighted in purple, sky blue, and forest green, respectively. (**B**) Ribbon diagram of the simian VP7 protein (PDB ID: 3FMG [48]). The Rossman fold domain is colored yellow, and the beta barrel domain is colored orange, as in the original paper (48). The surface is rendered at 80% transparency. The coloring of the shaded regions in the epitope regions is the same as in panel A. The side chains of the residues involved in the epitope regions are depicted using stick rendering. Residues that are identical between simian VP7 and equine G3 VP7 but different from equine G14 are indicated with sky blue colored asterisks (seven residues across all three epitope regions: N94, N96, A125, and V129 in 7-1a, T147 and A221 in 7-1b, and D211 in 7-2). A single residue identical between simian VP7 and equine G14 VP7 but different from equine G3 is indicated by a red asterisk (D145 in 7-1b). (**C**) Analysis of VP7 among ERVA strains currently circulating in central Kentucky (G3; *n* = 5 and G14; *n* = 4), reference ERVA strain Eq ref G3P[12], reference Eq ref G14P[12], and the vaccine H2 strain. Field isolates CO2024 ID110, NY2022 ID46, LA2022 ID51, LA2024 ID101, and TX2024 ID97 are not shown as they are completely identical. Antigenic residues are located in regions 7-1a, 7-1b, and 7-2. Amino acid residues that are identical in both SA11 G3P[2] and Eq G3P[12] but differ from Eq G14P[12] are highlighted in green. Residues that are identical in both equine strains (Eq G3P[12] and Eq G14P[12]) but differ from SA11 G3P[2] are highlighted in red.

The rSA11 G3 and the equine G3 strain share a 94.2% amino acid sequence identity in the VP7 protein, with 19 amino acid differences. In contrast, rSA11 G3 and equine G14 have a lower similarity of 88.7% in the VP7 protein, with 37 amino acid differences. The greatest divergence in VP7 protein among these three viruses is between the two equine strains, G3 and G14, which share 88.0% homology, differing at 39 amino acid positions. To better understand the cross-neutralization patterns that were observed above, we compared the VP7 antigenic sites of rSA11 G3, equine Eq-ref-G3P[12], and Eq-ref-G14P[12] strains (Fig. 7A) and mapped the epitope regions onto the simian VP7 crystal structure (PDB ID: 3FMG) (Fig. 7 and Table 1). Since rSA11 G3P[2] and equine G3P[12] share the same G genotype, the majority of their VP7 epitope residues are conserved, unlike the G14 strain, which belongs to a different G genotype. In the 7-1a epitope region, which occurs in the Rossman fold domain (Fig. 7A and B), 5 of 14 epitope residues (N94, N96, A125, V129, and A130) were identical between rSA11 G3 and equine G3 but differed in G14 (Fig. 7A and C). Similarly, the 7-1b domain contained a common D211 residue in both rSA11 G3 and equine G3, which was different in the G14 VP7 sequence. In the 7-2 epitope region, found in the beta barrel domain, residues T147 and A221 were conserved between rSA11 G3 and equine G3 but differed in G14 (Fig. 7A and B). These shared antigenic sites may contribute to the structural similarity and explain the comparable and high neutralization responses observed between rSA11 G3 and equine G3, but not with G14. In contrast, only a single residue D145, which occurs in the Rossman fold domain of the 7-2 epitope region (Fig. 7A and B), was conserved between rSA11 and equine G14, but differed from equine G3 (Fig. 7A), and this unique residue is highlighted on the structure with a red asterisk (Fig. 7B).

**TABLE 1:** Comparison of amino acid antigenic epitopes among strains, including identity, percentage similarity, and mismatches

Protein	Comparison	% Identity	% Similarity	Mismatches
VP7	SA11 G3P[2]^*a*^ vs Eq G3P[12]^*b*^	307/326 (94.2%)	315/326 (96.6%)	19
	SA11 G3P[2]^*a*^ vs Eq G14P[12]^*c*^	289/326 (88.7%)	310/326 (95.1%)	37
	Eq G3P[12]^*b*^ vs Eq G14P[12]^*c*^	287/326 (88.0%)	310/326 (95.1%)	39
VP4	SA11 G3P[2]^*d*^ vs Eq G3P[12]^*e*^	644/776 (83.0%)	719/776 (92.7%)	132
	SA11 G3P[2]^*d*^ vs Eq G14P[12]^*f*^	638/776 (82.2%)	717/776 (92.4%)	138
	Eq G3P[12]^*e*^ vs Eq G14P[12]^*f*^	759/776 (97.8%)	769/776 (99.1%)	17
VP8*^g^	SA11 G3P[2]^*d*^ vs Eq G3P[12]^*e*^	105/161 (65.2%)	132/161 (82.0%)	56
	SA11 G3P[2]^*d*^ vs Eq G14P[12]^*f*^	101/161 (62.7%)	131/161 (81.4%)	60
	Eq G3P[12]^*e*^ vs Eq G14P[12]^*f*^	154/161 (95.7%)	157/161 (97.5%)	7

^*a*^ GenBank accession number LC178569.

^*b*^ GenBank accession number PP453583.

^*c*^ GenBank accession number PP453594.

^*d*^ GenBank accession number LC178567.

^*e*^ GenBank accession number PP453578.

^*f*^ GenBank accession number PP453589.

^*g*^ The asterisk (*) indicates amino acid positions 64–224 based on GenBank accession number.

### Sequence homology and structural insights into VP4 antigenic sites of rSA11 G3P[2], equine G3P[12], and G14P[12] genotypes

An overall VP4 sequence analysis indicated an 83% amino acid homology between the rSA11 G3P[2] and Eq G3P[12] strains, with 132 amino acid differences. Between rSA11 G3P[2] and Eq G14P[12], the sequence homology was 82.2%, with 137 mismatches. The sequence homology between Eq-ref G3P[12] and Eq-ref G14P[12] was 97.8%, with 17 amino acid mismatches (Table 1). Focusing on the VP8* domain, the sequence identity dropped to 65.2% between rSA11 G3P[2] and equine G3P[12], and to 62.7% between rSA11 P[2] and equine G14P[12], with 56 and 60 amino acid differences, respectively. In contrast, the two equine strains share 95.7% identity in VP8*, differing at just seven residues.

**Fig 8:**
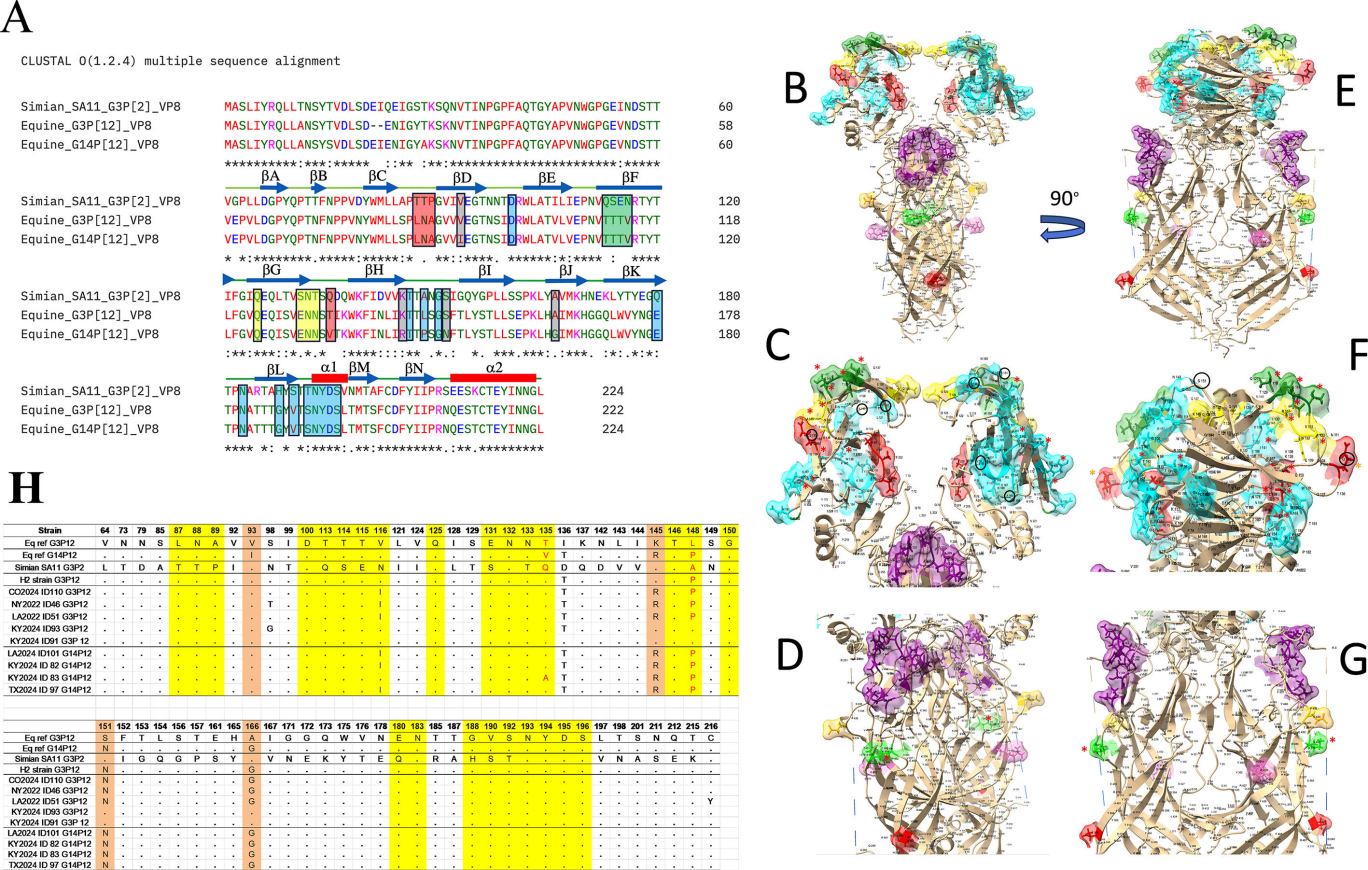
Mapping of VP5* and VP8* epitopes onto the structure of VP4. (**A**) Clustal (Omega) multiple sequence alignment using the three sequences Simian SA11-VP8, Eq-ref-G3P[12]-VP8*, and Eq-ref-G3P[14]-VP8*. (**B**) The cryo-EM structure of simian VP4 (PDB ID: 9c1I, front view) with the epitope regions colored as follows: 8-1, sky blue; 8-2, forest green; 8-3, yellow; 8-4, red; 5-1, purple; 5-2, yellow; 5-3, green; 5-4, hot pink; and 5-5, red. Epitope amino acid side chains are depicted as sticks, and the surface is rendered at 70% transparency. (**C**) Zoomed-in view of the VP8* receptor-binding domain showing 8-1 through 8-5 epitope regions. (**D**) Zoomed-in view of the VP5* domain showing 8-1 through 8-5 epitope regions. (**E**) Side view of the cryo-EM structure depicted in panel A rotated by 90°. Panels F and G are the same as panels C and D but rotated by 90°. In panels C, D, F, and G, residues that are the same between equine G3 and G14 but different from the simian epitope region are indicated by red asterisks. Residues that are different between all three are indicated by orange asterisks. Residues in VP8* that are the same between simian and equine G3 but different from equine G14 are indicated by black-lined circles. (**H**) Analysis of VP8 (64–224 aa) among ERVA strains currently circulating in the United States (G3; *n* = 5 and G14; *n* = 4), reference ERVA strain Eq ref G3P[12], reference Eq ref G14P[12], and the vaccine H2 strain. Antigenic residues in VP8 are highlighted in yellow VP8* (8-1 [aa 100, 146, 148, 150, 188, 190, 192–196], 8-2 [aa 180, 183], 8-3 [aa 113–116, 125, 131–133, 135], and 8-4 [aa 87–89]), and unique residues in antigenic sites at positions 135 and 148 in all strains are shown in red. Amino acid residues that are identical in both strains rSA11 G3P[2] and Eq ref G3P[12] at non-antigenic sites but differ from Eq ref G14P[12] are highlighted in orange.

To provide a structural insight into the observed differences and similarities in the virus neutralization properties between the three strains, the sequence similarities and differences of the three VP4 sequences were evaluated within the known antigenic determinant regions, which are the target of virus-neutralizing antibodies. The sequence comparison and analysis of the VP8* domain are highlighted in Fig. 8A. All of the previously identified antigenic regions of the VP4 protein were mapped onto the simian CryoEM fitted structure of the VP4 protein (EMDB ID: 45120; fitted PDB ID: 9c1I) in Fig. 8B through G. The amino acid sequence alignment of the VP4 gene segment revealed a clear divergence between simian and ERVAs, particularly within the VP8* antigenic sites (Fig. 8A through G). The simian strain had multiple substitutions compared to both equine strains, and unique substitutions were found at positions A148 and Q135, highlighted in red in Fig. 8H, C, and F. These sites are potentially important for differences in antibody-based neutralization among equine strains, since VP8* from equine Eq-ref-G3P[12] and Eq-ref-G14P[12] differ only at residues A148 and Q135. Despite the diversity among VP8* epitope sites, we noticed a stretch of conserved residues from 193 to 196 that could also contribute to a similar neutralization response even across diverse P genotypes. Notably, the antigenic regions within VP5* (5-1 to 5-5) were highly conserved between the equine strains, with only two amino acid differences (positions 135 and 148) within VP8* antigenic sites (Fig. 8B through H).

### Solvent accessibility of VP7 and VP8* amino acid residues

**Fig 9:**
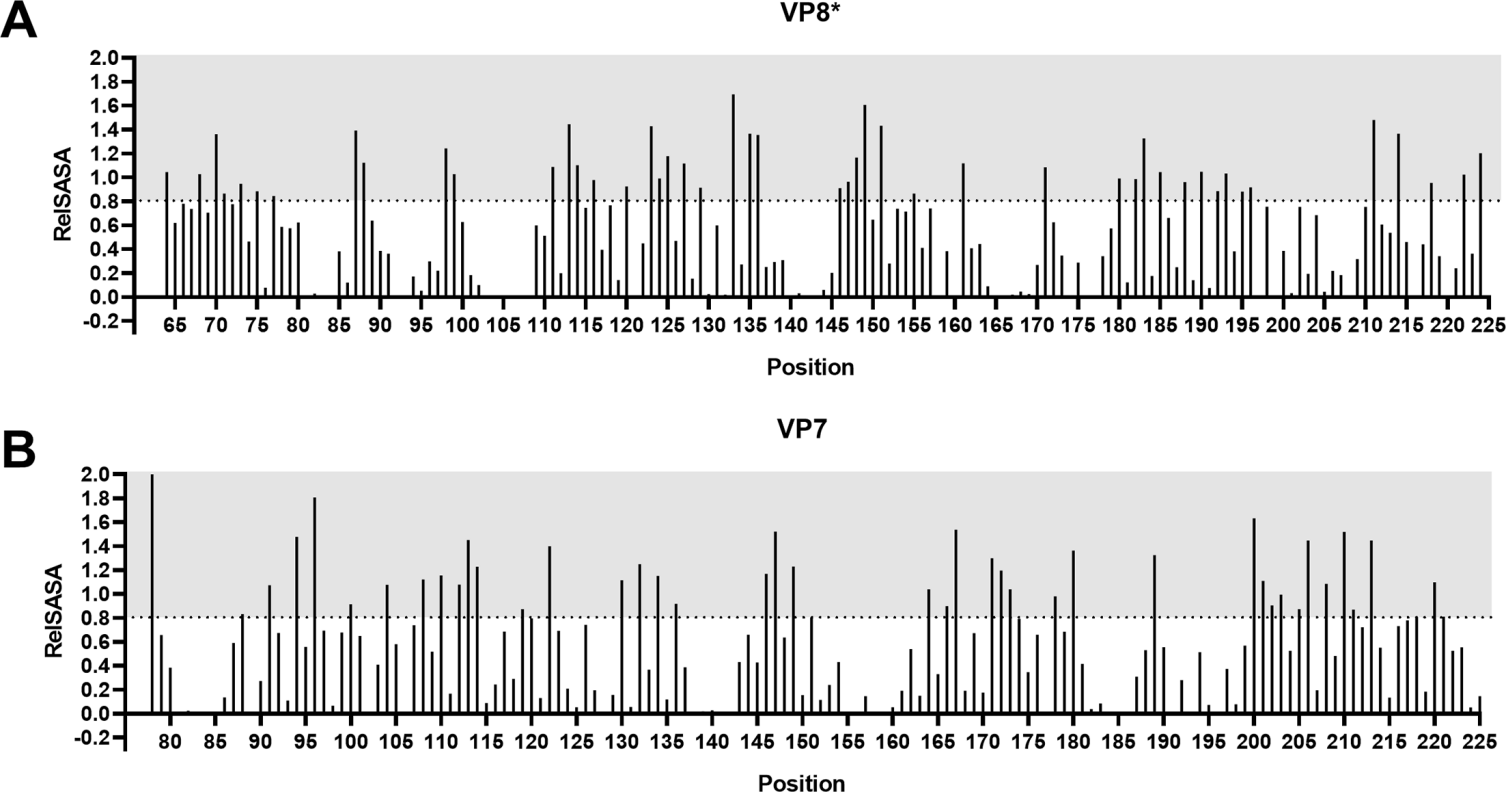
Relative solvent-accessible surface area analysis of rSA11. RelSASA values for (**A**) VP8* (residues 64–224) and (**B**) VP7 (residues 78–312) of the rSA11 strain were calculated to assess the surface exposure of amino acid residues**.** Signal peptide sequences were excluded from the analysis. The dotted line indicates the cutoff for RelSASA values greater than 0.8.

The antigenic epitopes described above were identified based on human strains and characterized through human and mouse studies (49–51). It is still unknown whether horses or rabbits utilize the same antigenic sites for the stimulation of RV-specific antibody responses as those that have been identified in humans or mice. It is also unclear whether other domains in the VP4 and VP7 proteins are involved in forming antigenic epitopes that are recognized by anti-RV antibodies. To probe this question and further understand whether the observed amino acid differences discovered in our study are potentially structurally relevant for antibody recognition, we calculated the relative solvent-accessible surface area (RelSASA) of each residue in VP7 and VP8*. The calculated values for known epitopes were mostly close to 1, which represents high solvent accessibility and potential accessibility to NAbs. Validation of known epitope residues supports the utility of our approach for further analyzing other amino acids and relating them to potential roles in antigenicity. Interestingly, there were some additional residues outside the known epitope regions that also showed high RelSASA values and could potentially be accessible to neutralizing antibodies (Fig. 9A and B; supplemental material). For example, there are 29 sites that do not occur in the epitope regions of human strains identified through mouse and human studies (49–51) but displayed RelSASA values > 0.8, indicating strong surface exposure. In addition, common surface-exposed residues in strains rSA11 G3P[2] and Eq-ref-G3P[12] that do not occur in previously identified antigenic sites in VP8* but differ in Eq-ref-G14P[12] include V93, K145, S151, and A166. Among these, only site 151 showed a high RelSASA value, indicating the solvent exposure and suggesting that it may directly alter epitope composition and antibody binding (Fig. 9A) (File S1). Similarly, in VP8*, residues at positions 135 and 148, which carry unique mutations in all strains (Fig. 9A), showed high solvent exposure (RelSASA > 0.8). Even though the RelSASA values at residues 135 and 148 were similar across strains, the amino acid substitutions likely influence antibody recognition, supporting their role in modulating antigenic properties. In contrast, in VP7 residue D145, the RelSASA value is >0.8, which is the same for the D145N substitution.

## DISCUSSION

Although an inactivated monovalent G3P[12] vaccine is available to control outbreaks in horses, yearly ERVA outbreaks are still observed during the foaling season throughout the United States. In addition to posing a constant threat to the equine industry, ERVAs can be potentially zoonotic and contribute their segments to new strains that are capable of infecting and causing enteric diseases in humans. This scenario has been well demonstrated by RV outbreaks in children under the age of 2 years, which were caused by equine-like G3 RV strains (52–54). Since RVs are still a concern in developing and low-income countries, extensive efforts have been made in recent years toward developing safe and highly effective vaccines with broad protection (55–57). Equine monovalent and multivalent vaccines are commonly used in the equine population worldwide. For example, the H2 strain G3 monovalent vaccine used in the equine industry in central Kentucky induces cross-reactive neutralizing antibodies against the heterologous equine strain G14P[12]. However, their titers are generally two- to fourfold lower than those against the homologous G3P[12] strain in mare serum, colostrum, milk, and foal serum (3). Meanwhile, the multivalent equine vaccine, which has been mainly used in Argentina, contains the simian SA11 G3P[2] strain (35), which can provide broader protection, especially against different G genotypes.

VP4 and VP7 have been historically viewed as minor and major immunogens, respectively, for driving the immune responses against RVs in humans and animals. Despite antibodies targeting either VP4 or VP7 being protective against RV infections, the precise correlates of immune protection are still not well defined. Recent VP8*-based RV vaccine developments did not show any significant breakthroughs in protective efficacy (56–60). Previous studies have considered the VP7 epitopes as the major neutralizing antigen, as VP7 is one of the most abundant capsid proteins on the surface of an RV virion. Furthermore, VP7 elicited NAbs generally provide strong protection in animals (40, 45, 61, 62). On the other hand, VP4 is considered a secondary immunogen, though it is critical for receptor binding and host cell membrane penetration (63). A number of studies have previously shown that antibodies against VP4, particularly its VP8* receptor binding segments and VP5* domains, majorly contribute to cross-protection and disarm viral infectivity (57, 64). Despite extensive studies, there remains a significant gap in understanding the determinants of cross-neutralization among different RVs, which has presented a hurdle to the development of a universal RV vaccine that is able to provide durable and broad protection against multiple RV genotypes.

In this study, we investigated the potential value of including simian SA11 G3P[2] strain in ERVA vaccines, particularly focusing on better understanding of antibody responses mediated by the outer capsid protein VP7 and the spike protein VP4, in protecting cells from infection by different G and P genotypes represented by equine G3P[12] (i.e., sharing the same G genotype with SA11) and G14P[12] (i.e., possessing different G and P genotypes from SA11). Our virus neutralization experiments generated several new observations with respect to the determinants of cross-neutralization among RVs with different G and P genotypes. Despite the difference in P genotype between rSA11 G3P[2] and equine G3P[12], substantial VP7 antigenic conservation between different strains can mediate robust cross-neutralization between these two viruses sharing the same G genotype. By sequence and antigenic site analyses coupled with neutralization data, we demonstrated that both sequence similarity and specific epitope conservation strongly influence neutralization outcomes. The NAb response raised by similar G genotype strains, as expected, provided a stronger protective antibody response, which was not observed with responses raised by similar P genotype strains (45).

Robust cross-neutralization responses can occur among three different RVs analyzed in this study, even between rSA11 G3P[2] and equine G14P[12] with distinct G and P genotypes. Sequence analysis of rSA11 G3P[2] and equine G14P[12] revealed that 13 out of 37 amino acid mismatches in the VP7 protein are located in antigenic sites, and only site 145 showed similarity between simian G3 and equine G14 but differed in similar G genotype strains (i.e., rSA11 G3 and equine G3 strains). The additional amino acid similarity at position D145 between rSA11 and Equine G14 is notable. Structural mapping shows that residue D145 is located within an antigenic loop and is surface-exposed; this site can alter properties from hydrophobic to hydrophilic. Due to its surface-exposed position, this residue is considered highly important and is monitored in RV surveillance and vaccine studies, as it can reduce the neutralization response against animal strains (65–67). The presence of residue D145 introduces a potential N-linked glycosylation site near the 7-1a epitope (65, 68). Therefore, a single conserved residue at D145 between rSA11 G3 and equine G14 may contribute to structural similarity and explain the comparable and high neutralization responses observed between rSA11 G3 and equine G14, but not with G3. Even though the RelSASA values at residues 135 and 148 in VP8* and residue 145 in VP7 were similar across strains with different amino acid residues at these positions, they can still influence antibody recognition, supporting their importance in antigenic properties. Nevertheless, the substitution introduces no change in RelSASA-based measurement but creates differences in side-chain chemistry, such as hydrophobicity, polarity, or glycosylation, which may alter the local antigenic surface and contribute to reduced neutralization despite unchanged accessibility (69). Thus, both surface-exposed residues and side chains of residues together define cross-neutralization outcomes, explaining why some conserved accessible sites are immunodominant, whereas others with substitutions lose reactivity.

Intriguingly, high cross-neutralization was observed between rSA11 G3P[2] and equine G3P[12], despite changes in residues 123, 212, 213, and 146 in the VP7 antigenic site. It may indicate that either changes in these positions do not appear to influence the binding affinity of neutralizing antibodies or may still retain structural similarity. Moreover, we noticed a few additional amino acid similarities between rSA11 G3 and equine G14 VP7: residues R28, I29, F47, A181, V218, V233, I268, and V309. Though these residues are not part of the antigenic sites, they might influence protein folding or stability of antigenic loops and indirectly affect antibody binding. It should be noted that these findings require further experimental investigation to fully elucidate the importance of these residues and their impact on the immunogenicity of the RV strains.

The VP4 comparison further illuminates the relationship between structural conservation and neutralization breadth. Surprisingly, we found a difference in NAb responses between two equine strains having similar P[12] sequences with 95.7% homology. The only differences in the VP8* domain between these two strains were at residues 135 and 148, which could be important in immunogenicity and a possible reason for reduced neutralizing antibody response between them. Furthermore, we found that rabbit equine G3 VP8* serum showed a similar level of neutralizing antibody response between P[2] and P[12], despite the fact that these strains have a reduced percentage of amino acid identity in VP8*. We reason that despite the substantial divergence in VP8* epitope sites between these two strains, cross-reactive antibody responses appear to target epitopes conserved in VP8*. Since our conclusions are based on a truncated VP8* fragment (64–224 AA), whereas previous studies have shown that inclusion of the N-terminus of VP8* and the body and stalk regions of VP5*, such as 26–231 AA and 26–476 AA, can stimulate higher levels of neutralizing antibodies than VP8* and VP5* alone (70, 71), future studies including extended VP4 regions should be conducted to better understand the contribution of other VP4 domains such as VP5* to broadly heterotypic neutralization. To understand VP8* heterotypic responses, we examined amino acid similarities in VP8* (64–224 AA) between rSA11 G3P[2] and equine G3P[12] and the differences with equine G14P[12]. The residues 93, 145, 151, and 166 are identical in rSA11 G3P[2] and equine G3P[12], which may be important for the comparable antibody response. Furthermore, we also sequenced a few field equine isolates to confirm the conservation of these site mutations, and surprisingly, we noticed that the antibody raised against the G3 reference strain likely shows a similar response to the simian rSA11 strain due to high sequence identity at several key positions that are previously known for not playing any role in RV’s antigenicity, including V93, K145, S151, and A166. These conserved residues may contribute to the preservation of conformational epitopes, supporting cross-reactivity between the two strains. In contrast, the G14 reference strain differs at all four of these residues (I93, R145, N151, and G166), which may significantly alter the antigenic landscape and reduce the binding affinity of the G3-derived antibody. Although G3 field isolates show partial variability at residues 145, 151, and 166, the extent to which these substitutions affect antibody recognition remains uncertain. The G14 field isolates appear to be more conserved relative to the G14 reference; their antigenic reactivity with G3-derived antibodies cannot be definitively predicted without using the reverse genetics system coupled with functional neutralization or antibody-binding assays. Therefore, while the observed sequence conservation explains the equine antibody’s reactivity with the simian strain, further investigation is needed to assess cross-reactivity within diverse field isolates.

Our results are in agreement with earlier studies indicating that both VP7 and VP4 contribute to RV antigenicity but emphasize the predominant role of VP7 (G genotype) in stimulating cross-reactive neutralizing antibodies (45). These new data indicate a need for monitoring genetic polymorphisms in the VP7 protein of the field RV strains and relating whether these changes affect vaccine-mediated protection, which has been demonstrated in a recent study (65). This study established a functional link between a mutation found at position 145 of VP7 and vaccine breakthrough infection in children (65). The P genotype (based on VP8* sequence) is still considered important for vaccine design as it is crucial for receptor binding and host switching (57). Interestingly, our findings suggest that for equine strains, detailed epitope mapping in the context of equine antibodies is still required (57, 72–75). This is because, so far, epitope positions have only been elucidated based on human strains and may differ for equine strains. Furthermore, our data indicate that the precise epitope-level conservation can predict cross-protection better than overall sequence identity. This notion has important implications for the design of the next-generation RV vaccines, particularly those based on subunit or reassortant virus platforms. As shown here, inclusion of conserved VP7 epitopes could extend protection across multiple G genotypes, even in the presence of divergent VP4 segments or P genotypes.

Finally, our results demonstrate that different host species may exhibit functional differences in B-cell repertoire, antibody affinity, and isotype diversity, which can significantly influence the breadth and strength of cross-neutralizing antibody responses (76, 77). In contrast to rabbit sera, we observed that horse sera showed better cross-neutralization among different equine strains. This suggests that host species may have their own selective way for dominant epitopes and antibody specificity (78). Horses, being the natural host for equine RVs, may provide broadly cross-reactive antibodies, while rabbits, as non-natural hosts, may focus primarily on the variable epitopes and, as a result, produce narrower antibody responses. These observations highlight the importance of host species in defining the magnitude and breadth of neutralizing antibody responses. This finding has significant implications for vaccine development and preclinical testing, underscoring the need to evaluate candidate vaccines across multiple species or in species-relevant models to ensure broad and effective protection. One limitation of our study was that neutralization was assessed using the whole virus strains. Future investigation involving individual proteins and reverse genetics systems with mutagenesis at specific positions with an antigenic site would give a more precise understanding of epitope-based immune responses. In conclusion, the results of our experiments demonstrate that the determinants for cross-neutralization among different RVs are complex, which are likely dependent on the context (i.e., the combination of G and P genotypes) and animal species. Nevertheless, findings reported in this study offer novel insights into protective antibody responses against different RVs and suggest new strategies for broadening vaccine coverage across genetically distinct RV strains.

## MATERIALS AND METHODS

### Cell culture

African green monkey kidney epithelial cell line (MA-104) was grown in Dulbecco’s modified Eagle medium (DMEM) (Gibco, Invitrogen, USA) supplemented with 10% fetal bovine serum (FBS) (Gibco, USA) and 1× penicillin-streptomycin (Life Technologies, Carlsbad, CA, USA). An African green monkey kidney epithelial cell line defective in STAT1 and IRF3 response (MA104 N*V) was obtained from the University of Nottingham. The cells were generated in-house at the University of Nottingham, and a detailed description of the generation of this cell line was provided in the published article and dissertation (79, 80). MA104 N*V cells were propagated in DMEM supplemented with 10% FBS, 1× penicillin-streptomycin, and 3 µg/mL each of puromycin (Gibco, A1113803) and blasticidin (Gibco, A1113903). BHK cells stably expressing T7 RNA polymerase (BHK-T7) were used for simian virus generation (81). The BHK-T7 cell line was a kind gift from Ulla Buchholz at NIAID through John Patton, Indiana University. The cells were grown in DMEM supplemented with 5% FBS, 1× penicillin-streptomycin, 1× non-essential amino acids (Gibco), and 2% G418 included every alternate passage (Geneticin, Invitrogen).

### Plasmids

SA11 G3P[2] strain T7 plasmids were kindly provided by Takeshi Kobayashi through the Addgene plasmid repository GenBank accession numbers LC178564–LC178574 (https://www.addgene.org/Takeshi_Kobayashi/) (pT7-VP1SA11, pT7-VP2SA11, pT7-VP3SA11, pT7-VP4SA11, pT7-VP6SA11, pT7-VP7SA11, pT7-NSP1SA11, pT7-NSP2SA11, pT7-NSP3SA11, pT7-NSP4SA11, and pT7-NSP5SA11). An additional plasmid (pCMV/NP868R), which expresses the African swine fever virus NP868R capping enzyme, was used for virus rescue. VP8* from the Eq-ref-G3P[12] strain (190–672 nt; 64–224 AA) was amplified (primer sequences are available on request) by reverse transcription-PCR (RT-PCR), cloned into the pET-28a-His-tag vector. Following the purification, purified VP8*-P[12] protein was used to generate rabbit anti-G3P[12]-VP8* antiserum through a contract service to Pacific Immunology, Ramona, CA, USA.

### Preparation of ERVA clinical samples

ERVA genotype G3P[12] or G14P[12] RT-PCR-positive fecal swabs were collected from diseased foals. Fecal swabs were resuspended in PBS buffer and subjected to multiple freeze-thaw cycles. Samples were clarified by centrifugation at 3,000 rpm for 10 min, followed by filtration of the supernatant through a 0.45 µm membrane filter. A 500 µL aliquot of the processed samples was used to isolate equine RVs as previously described (8). For replicating the virus and making a long-term virus stock, following the virus isolation and sequence confirmation, the viruses were activated using 10 µg/mL TPCK-trypsin for 1 h at 37°C. After incubation, serial dilutions of the viruses were prepared and added to washed and serum-starved MA104 N*V cells. The infected plates were incubated at 37°C for 3–7 days and observed daily for cytopathic effects (CPEs). Infected cells underwent repeated freeze-thaw cycles, and the entire process was repeated several times to generate sufficient virus titers for the experiments.

### Generation of recombinant SA11

Recombinant simian SA11 G3P[2] virus (rSA11 G3P[2]) was generated using a modified plasmid-based reverse genetics system, as previously described (81). In short, T7-BHK cells were seeded in a 12-well plate (2 × 10^5^ cells/well) without G418-containing media. At 90% cell confluency, the cells were used for transfection. For transfection, the plasmid and transfection mixture were prepared. The mixture contained 100 µL of prewarmed Opti-MEM (Gibco), 0.8 µg of each of the nine pT7 plasmids, whereas 2.4 µg (three times more) of pT7-NSP2SA11 and pT7-NSP5SA11 were used. Additionally, 0.8 µg of the pCMV/NP868R plasmid was included, along with 25 µL TransIT-LT1 (Mirus; 2 µL per microgram of plasmid). The mixture was incubated for 20 min at room temperature. After incubation, the mixture was added dropwise to washed T7-BHK cells containing 1.2 mL Opti-MEM, and the cells were incubated at 37°C for 48 h. After incubation, the transfected cells were overlaid with 5 × 10^4^ MA-104 cells along with a final concentration of 0.5 µg/mL TPCK in the media, and the cells were further incubated for 3 days. After 3 days, the cells were freeze-thawed, and the lysate was collected, activated with 10 µg/mL TPCK, and transferred to fresh MA104 cells with 0.5 µg/mL TPCK and incubated until CPE was observed. After complete CPE, the virus was harvested and characterized for further use.

### Antibodies

Recombinant SA11 G3P[2] virus-specific rabbit sera were generated by Pacific Immunology, Ramona, CA, USA. Heat-inactivated rSA11 G3P[2] virus with a 1 × 10⁸ focus-forming unit (FFU)/mL titer was used to immunize two rabbits. The immunization involved one dose with Complete Freund’s Adjuvant, followed by an additional three doses with Incomplete Freund’s Adjuvant with 2-week intervals. Terminal bleeds and pre-immune rabbit sera were used for the immunofluorescence assay (IFA) as well as for the neutralization assay. Rabbit anti-G3P[12]-VP8 antiserum was generated by immunization with purified VP8* residues 64–224 of RV VP4. Purified VP8* was prepared in a Tris buffer (20 mM Tris, 250 mM NaCl, and 1% glycerol, pH 8.0) with a final concentration of 1.5 mg/mL. Two rabbits were immunized with a total of four immunizations per animal, with approximately 150–200 µL administered per dose containing 225–300 µg VP8* protein, and pre-bleed and final production bleeds were collected over a 13-week immunization period. Equine hyperimmune sera to SA11 G3P[2] serum was provided by Peter Timoney from the University of Kentucky. Rabbit polyclonal serum was generated by Covance (Labcorp) against the representative (reference) strains ERVA G3P[12] and G14P[12] that had been described previously (8). These strains (GenBank accession numbers PP453575–PP453596) were previously described as RVA/Horse-tc/USA/KY-0809N/2021/G3P[12] and RVA/Horse-tc/USA/KY-0316FCL/2021/G14P[12], respectively (8).

### Virus neutralization assay

We conducted a fluorescent focus neutralization (FFN) assay for the rapid evaluation of neutralizing antibody responses against equine and simian RVs in this study. Briefly, confluent monolayers of MA104 N*V cells were seeded in 96-well plates and used for neutralization experiments. A total of 300 FFU of trypsin-activated virus, diluted in a total of 50 μL plain media, was incubated with 50 μL of twofold serially diluted rabbit or horse polyclonal serum, resulting in a final volume of 100 μL for the serum-virus mixture. The mixture was incubated at 37°C for 1 h. Following incubation, the mixture was transferred to the cell monolayers in the 96-well plate and incubated for an additional hour. After incubation, the mixture was discarded, cells were washed once with PBS buffer, and media containing 0.5  μg/mL TPCK were added. Plates were then incubated for 18 h at 37°C. After incubation, the media were discarded, cells were fixed, and IFA was performed. The highest dilution of the sera showing 50% reduction in fluorescent foci compared to serum negative controls was the endpoint neutralization titer. Serum samples with an FFN titer less than 20 will be scored as negative. Each assay was performed in duplicate, and each experiment was repeated three times.

### Foal vaccination

The licensed Zoetis monovalent G3 ERVA vaccine—the inactivated cell-culture-adapted H2 (G3P[12]) strain, RVA/Horse-tc/GBR/H-2/G3P[12]/1976 (82)—was intramuscularly injected into pregnant mares at 8, 9, and 10 months of gestation. Protective immunity is passed to the foals through the colostrum. However, in our experiments, mares were not vaccinated during gestation; instead, the three foals were off-label vaccinated at 2 and 3 months of age (Fig. S3). Foals were housed at the University of Kentucky research farm, and the herd consisted of mixed-breed horses of various ages. On this farm, horses are not routinely vaccinated against ERVA, and during the year of the study, no foals were diagnosed with ERVA. Serum was collected before vaccination of the foals (Foal X004, Foal X005, and Foal XX8). Foals were vaccinated with 1 mL of the Zoetis monovalent G3 ERVA vaccine intramuscularly and were monitored for any adverse reactions. Two control foals were injected at 2 and 3 months of age intramuscularly with 1 mL of sterile saline. Neither of the control foals showed an increase in virus neutralization titers for Eq-ref-G3P[12] and Eq-ref-G14P[12] at 7 days after the second vaccination (titer ≤64 and 16, respectively). There was no ERVA detected on the research farm in the year of the study. Serum was collected from the foals 7 days post-first (D7-Dose 1) and 7 days post-second vaccination (D7-Dose 2).

### Immunofluorescence assay

Media were discarded from virus-infected MA104 N*V cell monolayers, and cells were washed once with 1× PBS, followed by fixation in 80% acetone. After fixation, cells were stained with a 1:200 dilution of the primary antibody (goat anti-RVA polyclonal antibody) for 1 h, followed by washing twice with 1× PBS and incubated with a 1:400 dilution of the secondary antibody (rabbit anti-goat IgG Alexa Fluor 488) for 1 h each. Cells were visualized and quantified by counting fluorescent foci units under a fluorescence microscope using a Nikon Ti-E Eclipse fluorescence microscope.

### Structure models and identification of the antigenic site differences in VP4 and VP7 proteins of equine and simian RVs

The simian RV VP7 protein structure was used for epitope mapping and analysis (PBD ID: 3FMG [48]). The antigenic site sequence variations among equine and simian RV sequences were analyzed using a Clustal (Omega) sequence alignment (83). For RVA, a nucleotide identity cutoff range of 84%–89% is used for both VP4 and VP7 to classify P and G genotypes (15). The epitope sites were mapped onto the VP7 structure based on previously reported sites identified in human RVs (68). The Clustal (Omega) sequence alignment was performed using the human reference RV sequence used to identify the epitope sites; these sites were then highlighted in the sequence alignment and mapped onto the protein structure. The VP7 antigenic sites are divided into two defined epitopes: 7-1 and 7-2. The 7-1 epitope is further divided into 7-1a and 7-1b. For the VP8* and VP5* analysis, the cryo-EM-fitted structure of simian VP4 protein was used (EMDB ID: 45120; fitted PDB ID: 9c1I). The antigenic sites in the VP4 protein were divided into four predefined epitopes in VP8* (8-1, 8-2, 8-3, and 8-4) and five epitopes in VP5* (5-1, 5-2, 5-3, 5-4, and 5-5) (68).

### RT-PCR and genome sequencing

RNA was extracted from ERVA-positive samples using the QIAamp Viral RNA Mini Kit (Qiagen, 52904) according to the manufacturer’s instructions. Briefly, viruses were lysed, RNA was washed multiple times, and the final RNA product was eluted. The concentration of the extracted RNA was then determined by nanodrop, and RT-qPCR was performed to confirm the ERVA genotype in the final RNA product.

Sequencing was done using the Illumina MiSeq machine and Illumina reagents: Illumina Stranded Total RNA Prep, Ligation with Ribo-Zero Plus (Illumina, 20040525), and MiSeq Reagent Kit version 3 (600 cycles; Illumina, MS-102-3003). RNA preparation was done according to the manufacturer’s instructions as described in the Illumina Stranded Total RNA Prep, Ligation with Ribo-Zero Plus Reference Guide. Briefly, 100 ng of input RNA was used, which was depleted of ribosomal RNA, fragmented, and denatured. Then, cDNA was synthesized, 3′ ends were adenylated, anchors were ligated, the fragments were cleaned up, and finally, the library was amplified and cleaned up. The quantity and quality of the libraries of the individual samples were measured by TapeStation, and the total input of each sample into the machine was adjusted accordingly.

Sequences were assembled using the reference genomes, and whole genome single-nucleotide polymorphism analysis was performed. Briefly, the raw data from the MiSeq machine were then analyzed through an automated pipeline using the University of Kentucky’s Morgan Compute Cluster. This pipeline included FastQC quality control, trimming of reads through Trim Galore, indexing and alignment through BWA, sorting, indexing, and Flagstat using SAMtools, variant calling and consensus sequence generation done by BCFtools, and amino acid sequence generation through SeqKit.

### Calculation of relative solvent-accessible surface areas

The RelSASAs were calculated manually using the following procedure. The PDB file of interest (PDB ID: 3FMG for VP7 or PDB ID: 9c1I for VP8*/VP5*) was opened using the ChimeraX software package (84–86). The solvent-accessible surface area (SASA) for the individual amino acids in the structure was determined using the “measure sasa” command, and the resulting SASA values were saved in a file called “myfilename.defattr.” A file containing the solvent-excluded surface areas for all 20 amino acids (representing the reference maximum SES areas for each amino acid in a “gxg” reference context) was downloaded from the ChimeraX site (https://www.cgl.ucsf.edu/chimera/docs/ContributedSoftware/defineattrib/areaSESgxg.txt). The SASA and SESgxg values were imported into an Excel file. The RelSASA values were computed by dividing the SASA values by the SESgxg reference values and multiplying by 100 to yield the percent SASA relative to the maximum reference values. RelSASA values > 20% were considered solvent exposed.

## Data Availability

The genome sequences were deposited at NCBI. Accession numbers for the VP4 segments are as follows: CO2024 ID110 (PX460260), NY2022 ID46 (PX460256), LA2022 ID51 (PX460257), KY2024 ID93 (PX460259), KY2024 ID91 (PX460258), LA2024 ID101 (PX460264), KY2024 ID82 (PX460261), KY2024 ID83 (PX460262), and TX2024 ID97 (PX460263). Accession numbers for the VP7 segments are KY2024 ID93 (PX460253), KY2024 ID91 (PX460252), KY2024 ID82 (PX460254), and KY2024 ID83 (PX460255). The solvent-excluded surface definition file used for structural analyses was obtained from the ChimeraX website and is publicly available at https://www.cgl.ucsf.edu/chimera/docs/ContributedSoftware/defineattrib/areaSESgxg.txt.

## SUPPLEMENTAL MATERIAL

Fig. S1 jvi.00199-26-s0001.tif Neutralization antibody response in the prevaccinated serum panel derived from vaccinated foals.

Fig. S2 jvi.00199-26-s0002.tif Neutralization antibody response in the serum panel derived from vaccinated foals.

Fig. S3 jvi.00199-26-s0003.tif Schematic diagram illustrating the vaccination schedule, including immunization and serum collection time points.

Supplemental legends jvi.00199-26-s0004.docx Descriptive legends for Fig. S1 to S3.

Tables S1 and S2 jvi.00199-26-s0005.xlsx Relative solvent-accessible surface area (RelSASA) analysis for SA11 VP8* and VP7 values.

ASM does not own the copyrights to Supplemental Material that may be linked to, or accessed through, an article. The authors have granted ASM a non-exclusive, world-wide license to publish the Supplemental Material files. Please contact the corresponding author directly for reuse.
