# Supplementary figures and images for: Cross-neutralization and antigenic characterization of simian and equine group A rotaviruses
Source: J Virol. 2026 Mar 31;100(4):e00199-26. doi: 10.1128/jvi.00199-26 (PMC13098257; doi:10.1128/jvi.00199-26)

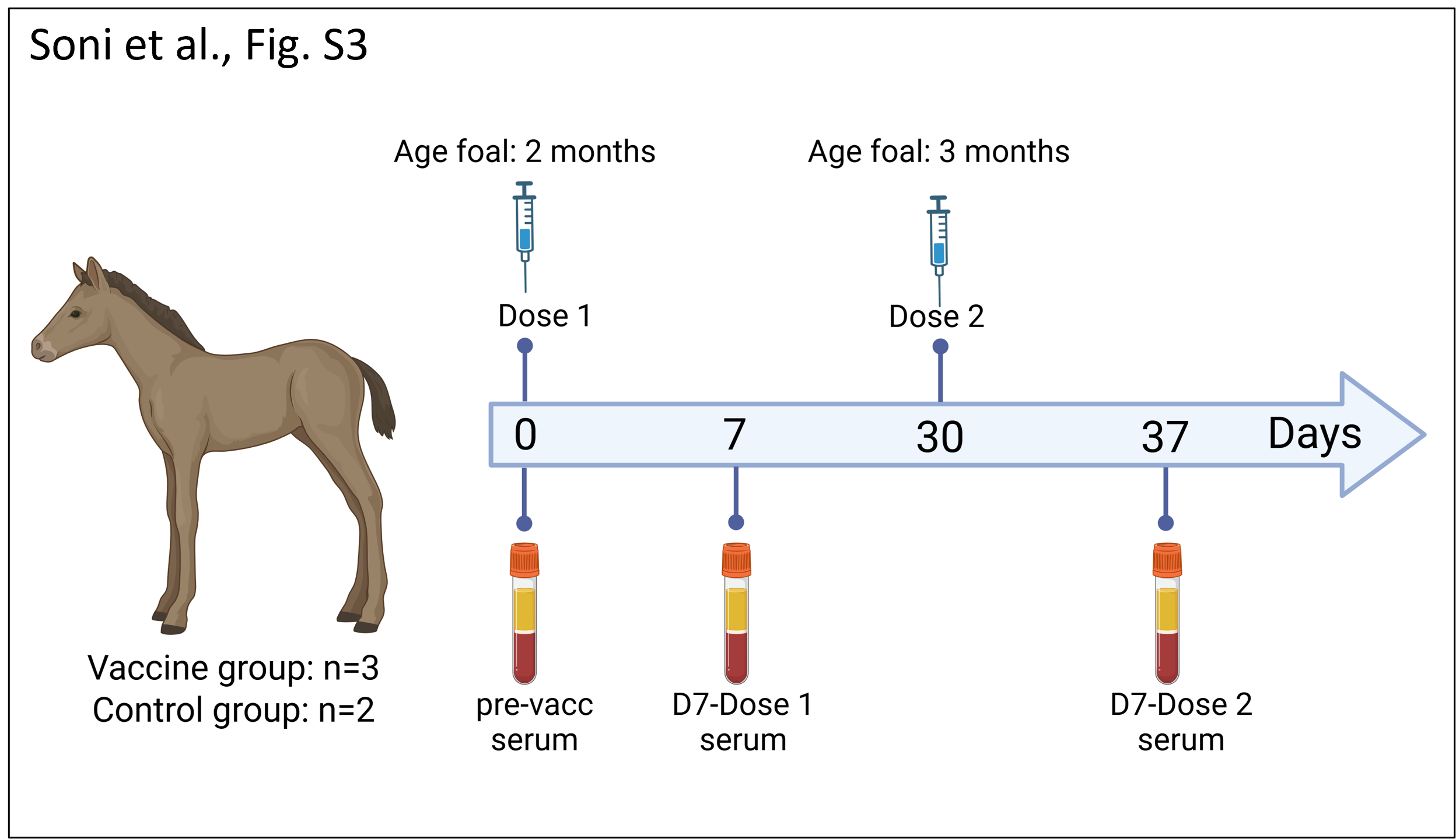

Supplement: Fig. S3 — Schematic diagram illustrating the vaccination schedule, including immunization and serum collection time points. [file jvi.00199-26-s0003.tif]
