# Supplementary material for: Cross-neutralization and antigenic characterization of simian and equine group A rotaviruses
Source: J Virol. 2026 Mar 31;100(4):e00199-26. doi: 10.1128/jvi.00199-26 (PMC13098257; doi:10.1128/jvi.00199-26)
Supplement: Supplemental legends — Descriptive legends for Fig. S1 to S3. [file jvi.00199-26-s0004.docx]

**Supplementary Figure Legends**

**Fig. S1. Neutralization antibody response in the pre vaccinated sera panel derived from vaccinated foals.** The serum samples from vaccinated foals, collected before vaccination (pre-vacc) were tested for their ability to neutralize RV strains: reference equine Eq-ref-G3P[12], and reference equine Eq-ref-G14P[12]. Neutralization titers are plotted on a logarithmic (log2) scale, while point represents the measurements from three independent experiments; horizontal bars indicate the mean ± standard deviation. Statistical analysis was performed using two-way ANOVA.

**Fig. S2. Neutralization antibody response in the sera panel derived from vaccinated foals.** The serum samples from vaccinated foals, collected seven days after second vaccinations, were tested for their ability to neutralize other demographic United state circulation strains: NY2022 ID46 G3P[12], LA2022 ID51 G3P[12], LA2024 ID101 G14P[12] and TX2024 ID104 G14P[12. Neutralization titers are plotted on a logarithmic (log2) scale, while point represents the mean of duplicate measurements from three independent experiments; horizontal bars indicate the mean ± standard deviation. Statistical analysis was performed using two-way ANOVA.

**Fig. S3. A schematic diagram illustrating the vaccination schedule, including immunization and serum collection time points.**
